# Chromatin Binding of *c*-REL and p65 Is Not Limiting for Macrophage *IL12B* Transcription During Immediate Suppression by Ovarian Carcinoma Ascites

**DOI:** 10.3389/fimmu.2018.01425

**Published:** 2018-06-27

**Authors:** Annika Unger, Florian Finkernagel, Nathalie Hoffmann, Felix Neuhaus, Barbara Joos, Andrea Nist, Thorsten Stiewe, Alexander Visekruna, Uwe Wagner, Silke Reinartz, Sabine Müller-Brüsselbach, Rolf Müller, Till Adhikary

**Affiliations:** ^1^Institute for Molecular Biology and Tumor Research (IMT), Center for Tumor Biology and Immunobiology (ZTI), Philipps University of Marburg, Marburg, Germany; ^2^Experimental Tumor Research Group, Center for Tumor Biology and Immunobiology (ZTI), Philipps University of Marburg, Marburg, Germany; ^3^Genomics Core Facility, ZTI, Philipps University of Marburg, Marburg, Germany; ^4^Institute for Medical Microbiology and Hygiene, Biomedical Research Center (BMFZ), Philipps University of Marburg, Marburg, Germany; ^5^Clinic for Gynecology, Gynecological Oncology and Gynecological Endocrinology, Philipps University of Marburg, Marburg, Germany; ^6^Clinic for Gynecology, Gynecological Oncology and Gynecological Endocrinology, ZTI, Philipps University of Marburg, Marburg, Germany

**Keywords:** IL12B, NFκB, REL, p65, ascites, ovarian carcinoma, macrophages, immunosuppression

## Abstract

Tumors frequently exploit homeostatic mechanisms that suppress expression of IL-12, a central mediator of inflammatory and anti-tumor responses. The p40 subunit of the IL-12 heterodimer, encoded by *IL12B*, is limiting for these functions. Ovarian carcinoma patients frequently produce ascites which exerts immunosuppression by means of soluble factors. The NFκB pathway is necessary for transcription of *IL12B*, which is not expressed in macrophages freshly isolated from ascites. This raises the possibility that ascites prevents *IL12B* expression by perturbing NFκB binding to chromatin. Here, we show that ascites-mediated suppression of *IL12B* induction by LPS plus IFNγ in primary human macrophages is rapid, and that suppression can be reversible after ascites withdrawal. Nuclear translocation of the NFκB transcription factors *c*-REL and p65 was strongly reduced by ascites. Surprisingly, however, their binding to the *IL12B* locus and to *CXCL10*, a second NFκB target gene, was unaltered, and the induction of *CXCL10* transcription was not suppressed by ascites. These findings indicate that, despite its reduced nuclear translocation, NFκB function is not generally impaired by ascites, suggesting that ascites-borne signals target additional pathways to suppress *IL12B* induction. Consistent with these data, IL-10, a clinically relevant constituent of ascites and negative regulator of NFκB translocation, only partially recapitulated *IL12B* suppression by ascites. Finally, restoration of a defective IL-12 response by appropriate culture conditions was observed only in macrophages from a subset of donors, which may have important implications for the understanding of patient-specific immune responses.

## 1 Introduction

Solid tumors are frequently accompanied by large numbers of tumor-associated macrophages (TAMs), and their abundance is correlated with poor prognosis in several tumor types (1, 2). Advanced ovarian carcinoma (OC) often coincides with considerable accumulation of a malignant peritoneal effusion termed ascites which harbors large numbers of floating stromal and tumor cells. Its immunosuppressive properties are at least in part conferred by soluble mediators (3, 4). Like their counterparts from other tumor entities, TAMs isolated from OC ascites display an anti-inflammatory phenotype including high expression of the hemoglobin scavenger receptor CD163 (5). In follow-up studies, we found that the transcriptomes of ovarian carcinoma TAMs and those of peritoneal macrophages from non-tumor patients are very similar with the exception of a set of genes involved in extracellular matrix reorganization (6), which is a hallmark of wound healing and tumorigenesis. The expression of this gene set is correlated with poor survival (6, 7). On the other hand, elevated expression of an interferon (IFN)-inducible gene set in TAMs is correlated with improved survival (7). A recent meta-analysis of TAM studies in OC patients (8) reflects the findings that CD163 expression correlates with worse prognosis, while pro-inflammatory macrophage polarization is positively associated with survival.

The ability of human macrophages to produce nitric oxide has been debated extensively (9–14). Apparently, their cytotoxic activity is rather exerted indirectly *via* secretion of cytokines, especially interleukin-12 (IL-12), which activates cytotoxic functions of T and NK cells (15–19). IL-10 is an immunosuppressive cytokine present in large amounts in OC ascites (20), and there is a strong negative correlation of its level with patient survival (5, 21). In the ovarian tumor microenvironment, *IL10* is expressed predominantly by TAMs (3, 21, 22). A critical function of IL-10 is to repress transcription of *IL12B* (23, 24), which encodes for the p40 subunit of IL-12 and IL-23 that is limiting for heterodimer formation. Antigen-presenting cells are the main producers of IL-12p40 (25). On the other hand, IL-12 represses *IL10* transcription. This reciprocal blockade, “the IL-10–IL-12 circuit” (26), is enforced by positive feedback of IL-10 and IL-12 production, respectively (27). Mechanisms involved in these positive feedback loops are interdependent upregulations of IL-10 and CD163 (28–30) or, *vice versa*, instigation of IFNγ production by T and NK cells upon exposure to IL-12 (31–33); in turn, IFNγ enables production of IL-12 by monocytic cells. Indeed, it was shown that IFNγ is capable of relieving suppression of IL-12 production by OC ascites (7, 34) and is, therefore, required for the pro-inflammatory feedback loop. Taken together, switching between IL-10 and IL-12 production can toggle between the anti-inflammatory, immunosuppressive Th2 state, and pro-inflammatory, tumoricidal Th1 activation (23). The large number of immunosuppressive TAMs relative to other hematopoietic cells in OC ascites provides them with a decisive, pro-tumorigenic role in the microenvironment which at least in part depends on efficient suppression of *IL12B* transcription (4).

*IL12B* transcription is induced cooperatively by STAT1 and the NFκB pathway (27). Stimuli that activate each of these have antitumorigenic effects *in vivo* (7, 35). A critical role for the IFNγ pathway in *IL12B* expression is highlighted by mutations that cause autosomal Mendelian susceptibility to mycobacterial disease (MSMD). These were reported to occur in seven genes: *IL12B, IL12RB1, IFNGR1, IFNGR2, IRF8, ISG15*, and *STAT1* (36–38). IFNγ, if present during macrophage differentiation in OC ascites *in vitro*, can override suppression of IL-12 production (7, 34). Therefore, gene regulation by IFNγ and STAT1 is generally functional in macrophages exposed to ascites. Moreover, pretreatment of macrophages with IFNγ can prime them for stimulus-dependent IL-12 production (39), indicating that an IFNγ-mediated effect can limit the amplitude of the response.

The NFκB transcription factor *c*-REL (from now on abbreviated as REL), which is predominantly expressed in hematopoietic cells (40, 41), is crucial for *IL12B* expression (42, 43). The requirement for NFκB is underscored by the finding that mutations in *NEMO/IKKG* which disrupt induction of *IL12B via* CD40-IKKγ cause X-linked MSMD (37). Concomitant with diminished nuclear localization of p65, enhanced expression of p50 was observed in murine TAMs. Nuclear p50 may form heterodimers that contribute to *Il12b* suppression, and in TAMs lacking p50, *Il12b* inducibility was restored (44). IL-10 was shown to impinge on NFκB-dependent signaling by preventing nuclear translocation of p65 in human (45, 46) and rat macrophages (47) and of Rel in a mouse macrophage cell line (43). In summary, the pro-tumorigenic and anti-inflammatory effect of ascites may depend on restrainment of NFκB function, and IL-10 may be required for this effect.

We speculated that ascites disables translocation and chromatin binding of NFκB effector transcription factors and, in consequence, induction of *IL12B* transcription. To test this hypothesis, the impact of ascites on the induction of *IL12B* by the NFκB transcriptional activators REL and p65 was investigated. Our data show that cell-free OC ascites suppresses *IL12B* expression by primary human macrophages upon stimulation with LPS and IFNγ *in vitro via* a reversible mechanism. Both REL and p65 translocation to the nucleus was strongly impaired by exposure to ascites. However, induction of *CXCL10* mRNA by LPS and IFNγ was unaffected in the presence of ascites. High-throughput sequencing approaches after enrichment of several chromatin marks were used to map putative regulatory regions of the REL target genes *IL12B* and *CXCL10*. In the presence of ascites, REL and p65 were recruited to the *IL12B* and *CXCL10* loci, indicating that their reduced nuclear levels are still sufficient for chromatin binding. Furthermore, IL-10 contributes to but is not sufficient for full suppression of *IL12B* expression by macrophages *in vitro*. These data implicate an additional suppressive mechanism, mediated by ascites-borne soluble factors, which acts upstream of *IL12B* transcription.

## 2 Materials and Methods

### 2.1 Ascites Collection and Isolation of TAMs From Ovarian Cancer Ascites

Ascites was collected from untreated high-grade serous ovarian carcinoma patients undergoing first-line surgery at the University Hospital Marburg. Informed consent was obtained from all patients according to the protocols approved by the institutional ethics committee. Mononuclear cells were isolated from ascites by Lymphocyte Separation Medium (Capricorn, no. LSM-A) density gradient centrifugation and subsequent enrichment by adherent cell positive selection in autologous ascites. Tumor-associated macrophages were directly harvested for chromatin immunoprecipitation, directly lysed for RNA isolation or cultivated in R5 medium (RPMI 1640 (Life Technologies, no. 61870044) with 5% (v/v) human AB serum (human serum type AB (male), (Sigma no. H4522)), and 1 mM sodium pyruvate (Sigma, no. S8636)) or in 100% cell-free autologous ascites for 1–2 days with or without recombinant 50 ng/ml IFNγ (from *E. coli*; Biomol, no. 51564) as indicated.

### 2.2 Isolation and Culture of Monocyte-Derived Macrophages

Buffy coats from healthy adult volunteers were kindly provided by the Center for Transfusion Medicine and Hemotherapy at the University Hospital Giessen and Marburg. Mononuclear cells were isolated from peripheral blood mononuclear cells from female healthy donors. Ficoll density gradient centrifugation was performed with Lymphocyte Separation Medium (Capricorn), and the cells were further purified by adherent cell positive selection of healthy donor monocytes. Monocyte-derived macrophages (MDMs) were generated from monocytes (6–12 days differentiation period) from healthy donors by cultivation in RPMI 1640 (Life Technologies, no. 61870044) with 5% (v/v) human AB serum (human serum type AB (male), Sigma no. H4522) and 1 mM sodium pyruvate (Sigma, no. S8636) (R5 medium) or in cell-free ascites from ovarian cancer patients, as indicated.

### 2.3 Cytokine Treatment

MDMs were stimulated with 100 ng/ml LPS (*E. coli* 0111:b4 L4391; Sigma) and 20 ng/ml recombinant human IFNγ (from *E. coli*; Biomol, no. 51564) or 20 ng/ml recombinant human IL-10 from HEK293 cells (Biomol, no. 97490) as indicated.

### 2.4 RNA Isolation and RT-qPCR

Total RNA from TAMs was extracted with TRIfast (Peqlab, no. 30-2020) or from MDMs with the NucleoSpin RNA kit (Macherey&Nagel, no. 740955) according to the manufacturer’s instructions. Complementary DNA synthesis was carried out with the iScript cDNA Synthesis Kit (Bio-Rad, no. 170-8891SP) according to the manufacturer’s instructions with 250–500 ng of purified RNA per sample. Quantitative PCR analyses were performed in three technical replicates per sample using ABsolute SYBR Green master mix (Thermo Scientific, no. AB-1158B) in Mx3000p and Mx3005 thermocyclers (Stratagene). The ribosomal protein 27, large subunit (*RPL27*) transcript was chosen for normalization after testing three housekeeping genes selected from our RNA-seq datasets with eight different TAM samples. RT-qPCR was carried out using the following primers: *RPL27*, AAAGCTGTCATCGTGAAGAAC and GCTGTCACTTTGCGGGGGTAG; *IL12B*, GCGAGGTTCTAAGCCATTCG and ACTCCTTGTTGTCCCCTCTG; *CXCL10*, AAGCAGTTAGCAAGGAAAGGTC and GACATATACTCCATGTAGGGAAGTGA. Raw data were evaluated with the Cy0 method (48) or the MxPro 4.01 software from Stratagene for Ct value calculation as indicated.

### 2.5 IL-12p40 ELISA

Concentrations of p40 in cell-free supernatants of cultured cells were determined in three technical replicates per sample using an ELISA kit from Biolegend (no. 430706) according to the instructions of the manufacturer.

### 2.6 Flow Cytometry Analysis of Macrophages

MDMs were stained with APC-labeled α-CD206 (BioLegend Cat #321110 RRID:AB_571885) and PE-labeled α-CD163 (eBioscience no. 12-1639-42) as described previously (5). Isotype control antibodies were from BD Biosciences, Miltenyi Biotech, and eBioscience. Cells were analyzed by flow cytometry using a FACS Canto II cytometer and FACSDiva software (BD Bioscience), and results were calculated as percentage of positive cells and mean fluorescence intensities (MFI).

### 2.7 Subcellular Fractionation

Subcellular protein fractionation was performed after washing cells twice with ice cold PBS (Sigma). Cell pellets were subsequently lysed in hypotonic cytosol extraction buffer L1 (5 mM PIPES pH 8.0, 85 mM KCl, 0.5% (v/v) NP40, protease inhibitor mix (Sigma, no. P8340) 1:1,000) for 20–40 min on ice. Lysates were collected and centrifuged for 5 min at 2,000×*g*, 4 °C. Cytosolic extract (CE) supernatants were collected, and nuclear pellets were washed once in L1 and subsequently lysed in RIPA buffer (10 mM Tris–HCl pH 7.5, 150 mM NaCl, 1% NP40 (v/v), 1% sodium deoxycholate (w/v), 1 mM EDTA), 1:1,000 protease inhibitor Mix (Sigma), 25 U/ml benzonase (Merck Millipore, no. 70746) for generation of nuclear extracts (NE) in a ratio of 5:1 of cytosol to nuclear extract.

### 2.8 Immunoblotting and Protein Quantification

Immunoblots were performed according to standard protocols using the following antibodies: α-*c*-REL polyclonal antibody (Cell Signaling Technology Cat #4727 RRID:AB_2178843); α-p65/RELA monoclonal antibody (Cell Signaling Technology Cat #8242 also 8242P, 8242S RRID:AB_10859369); α-LDH polyclonal antibody (Santa Cruz Biotechnology Cat #sc-33781 RRID:AB_2134947); α-acetyl-histone H3 polyclonal antibody (Millipore Cat #06-599 RRID:AB_2115283); α-β-actin (AC-15) monoclonal antibody (Sigma-Aldrich Cat #A5441 RRID:AB_476744); α-rabbit IgG, HRP-linked (Cell Signaling Technology Cat #7074 also 7074S, 7074V, 7074P2 RRID:AB_2099233), and α-mouse IgG, HRP-linked (Cell Signaling Technology Cat #7076 also 7076S, 7076V, and 7076P2 RRID:AB_330924). Imaging and quantification was done using the ChemiDoc MP chemoluminescence imaging system and Image Lab software version 5 (Bio-Rad).

### 2.9 RNA Interference

Small interfering RNA transfection was performed according to the manufacturer’s protocol using the TransIT-X2 reagent from Mirus (no. 6000) or the Viromer GREEN reagent (Lipocalyx, no. 230055). The following equimolar mixtures of three siRNA oligonucleotides each from Sigma were used for transfection of macrophages: *REL* SASI-Hs01-00064620, SASI-Hs01-00064621, SASI-Hs01-00064622. Set of four Upgrade ON-TARGETplus from Dharmacon was used for *RELA* (LU-003533-00-0002), containing four siRNA oligonucleotides. MISSION siRNA Universal Negative Control #2 from Sigma was used as a control siRNA (si-ctrl). Cells were harvested 48 h after transfection.

### 2.10 Generation of Murine Bone Marrow-Derived Macrophages, *Il12b* Quantitative RT-PCR, and Il-12p40 ELISA

BMDMs were generated by cultivation of 2 × 10^6^ bone marrow cells/well derived from wild-type and *Rel*^−/−^ mice in RPMI 1640 medium supplemented with 10% heat-inactivated FCS and 10 ng/ml macrophage colony-stimulating factor (PeproTech) in six-well plates. After one week of cell culture, purity was tested by FACS staining for CD11b. Subsequently, BMDMs were stimulated with either LPS alone (100 ng/ml) or with LPS (100 ng/ml) in combination with recombinant IFNγ (10 ng/ml). 24 h after stimulation of BMDMs, supernatants from cell cultures were harvested. Murine Il-12p40 was measured with an ELISA kit (BD Biosciences, no. 555165) according to the manufacturer’s protocol. Total RNA was extracted from BMDM-derived cell pellets using the High Pure RNA Isolation Kit (Roche, no. 11828665001). cDNA was synthesized using the RevertAid First Strand cDNA Synthesis Kit (Thermo Scientific, no. K1621). Gene expression was analyzed with a 7500 Fast Real-Time PCR System (Applied Biosystems). The gene expression of *Hprt1* was measured as an internal control. The following primer sets were used: *Hprt1*, CTGGTGAAAAGGACCTCTCG and TGAAGTACTCATTATAGTCAAGGGCA; *Il12b*, ATGTGTCCTCAGAAGCTAACCATC and CGTGTCACAGGTGAGGTTCACT.

### 2.11 Chromatin Immunoprecipitation and MIRA

After adherence selection in Greiner tissue culture flasks (no. 660175) in autologous ascites, TAMs were washed with PBS twice. MDMs were differentiated as indicated. Fixation was performed with 1% formaldehyde in PBS for 10 min at room temperature followed by quenching with 125 mM glycin for 5 min. Cells were washed with ice-cold PBS twice and harvested using a cell scraper. The pellet was lysed in hypotonic buffer L1 (5 mM PIPES pH 8.0, 85 mM KCl, 0.5% (v/v) NP40) with protease inhibitor mix (Sigma, no. P8340, 1:1,000) for 20–40 min on ice. Nuclei were resuspended in ChIP RIPA buffer (10 mM Tris–HCl pH 7.5, 150 mM NaCl, 1% NP40 (v/v), 1% sodium deoxycholate (w/v), 1 mM EDTA) supplemented with 1:1,000 protease inhibitor mix (Sigma), incubated on ice for 10–20 min and sheared with a Branson S250D Sonifier (Branson Ultrasonics) using a microtip in 1 ml aliquots in 15 ml conical tubes. 52 pulses of 1 s, 4 s pause, 20% amplitude were applied with cooling of the sample in an ice–ethanol mixture or in a 15 ml tube cooler (Active Motif, no. 53077). A 15 min 20,000×*g* supernatant was precleared with 10 µg of IgG coupled to 100 µl of blocked sepharose slurry (see below) for 45 min at 4 °C with agitation. IP was carried out with 300 µl of precleared chromatin, equivalent to 3–8 × 10^6^ cells. For MIRA, an aliquot of the sample was reverted as described below, purified on a Qiagen PCR purification column (no. 28106), and 250 ng of DNA were used according to the manufacturer’s instructions (Methylcollector Ultra, Active Motif, no. 55005). ChIP was performed and evaluated as described using 4 µg per sample of the following antibodies: IgG pool, (Sigma-Aldrich Cat #I5006 RRID:AB_1163659) or normal rabbit IgG (Cell Signaling Technology Cat #2729S RRID:AB_1031062); α-*c*-REL (Santa Cruz Biotechnology Cat #sc-70 RRID:AB_2178727 and Santa Cruz Biotechnology Cat #sc-71 RRID:AB_2253705, 1:1 mixture); α-p65/RELA (Diagenode Cat #C15310256 RRID:AB_2721009); α-p65/RELA (Cell Signaling Technology Cat #8242 also 8242P, 8242S RRID:AB_10859369); α-H3K27me3 (Diagenode Cat #pAb-069-050 also ENCAB000ARJ RRID:AB_2616049); α-H3K4me3 (Diagenode Cat #pAb-003-050 also ENCAB000BKU, C15410003-50, C15410003-10 RRID:AB_2616052); α-H3K27ac (Diagenode, Diagenode, Cat #C15410174, RRID:AB_2716835); α-H3K4me1 (Diagenode Cat #pAb-037-050, RRID:AB_2561054); α-H3K9me3 (Diagenode Cat #pAb-056-050, RRID:AB_2616051); α-H3K36me3 (Abcam Cat #ab9050, RRID:AB_306966); α-C/EBPβ (Santa Cruz Biotechnology Cat #sc-150, RRID:AB_2260363). For precipitation, a mixture of protein A and protein G sepharose (GE Healthcare life sciences, no. 1752800 and no. 1706180) was washed twice with ChIP RIPA buffer and blocked with 1 g/l BSA and 0.4 g/l sonicated salmon sperm DNA (Life Technologies no. 15632011) overnight. 50 µl of blocked bead slurry (1:1 volume ratio with liquid phase) were used per IP. Samples were washed once in buffer I (20 mM Tris pH 8.1; 150 mM NaCl; 1% (v/v) Triton X-100; 0.1% (w/v) SDS; 2 mM EDTA), once in buffer II (20 mM Tris pH 8.1; 500 mM NaCl; 1% (v/v) Triton X-100; 0.1% (w/v) SDS; 2 mM EDTA), twice in buffer III (10 mM Tris pH 8.1; 250 mM LiCl; 1% (v/v) NP40; 1% (w/v) sodium deoxycholate; 1 mM EDTA) on ice, and twice in Qiagen buffer EB (no. 19086) at room temperature. Immune complexes were eluted twice with 100 mM NaHCO_3_ and 1% SDS (w/v) under agitation. Eluates were incubated overnight at 65 °C after adding 10 µg of RNase A and 20 µg of proteinase K in the presence of 180 mM NaCl, 35 mM Tris–HCl pH 6.8, and 9 mM EDTA. An input sample representing 1% of the chromatin used per IP was reverted in parallel. Samples were purified using the Qiagen PCR purification kit according to the manufacturer’s instructions, except that DNA-binding lipids were removed by washing the matrix twice with pure methanol as described (49) previous to the final washing step with the buffer included in the kit. ChIP-qPCR was performed in three technical replicates per sample with the ABsolute SYBR Green master mix (Thermo Scientific, no. AB-1158B) in Mx3000p and Mx3005 thermocyclers (Stratagene) using the following primers: *IL12B* −1,200 bp CCATCCCTGCTCTCGACCT and GAAATCTGCGCCCGCCTAAA; *IL12B* TSS, AGTGCTTACCTTGCTCTGGG and TACCAGCAACAGCAGCAGAA; *IL12B* +20 kbp, ACGCCGCCCTAGAAGAAG and TCCCTTTCACCTTCTCTGGA; *CXCL10* −5,000 bp, AGCTGGTGCAGAATATGCCTT and CACTGTGAGCTCGGGGAATC; *CXCL10* TSS, GAAGTCCCATGTTGCAGACTC and AACAGTTCATGTTTTGGAAAGTGA. Data were evaluated using the MxPro 4.01 software from Stratagene for Ct value calculation. Relative recoveries were determined as percentage of input using a ΔCt method (50).

### 2.12 Library Preparation and High-Throughput Sequencing

Libraries were synthesized from 1–2 ng of genomic DNA using the MicroPlex kit (Diagenode, no. C05010011) according to the manufacturer’s instructions. Samples were sequenced on an Illumina Hi-Seq 1000 (single-ended, 50 bp).

### 2.13 Statistical Tests

Paired *t*-tests were used to calculate *p*-values.

### 2.14 Bioinformatics and Data Deposition

Mapping of ChIP-Seq reads and peak calling were carried out as described (49). Peaks were filtered for at least 30 deduplicated tags and a fold change (FC) over IgG of ≥2 (normalized total read counts). All genomic sequence and gene annotation data were retrieved from Ensembl revision 74. ChIP-seq data were deposited at ArrayExpress (no. E-MTAB-6297).

## 3 Results

### 3.1 Suppression of *IL12B* in Monocytic Cells From Ovarian Carcinoma Patients Is Mediated by Soluble Factors From Ascites

**Figure 1:**
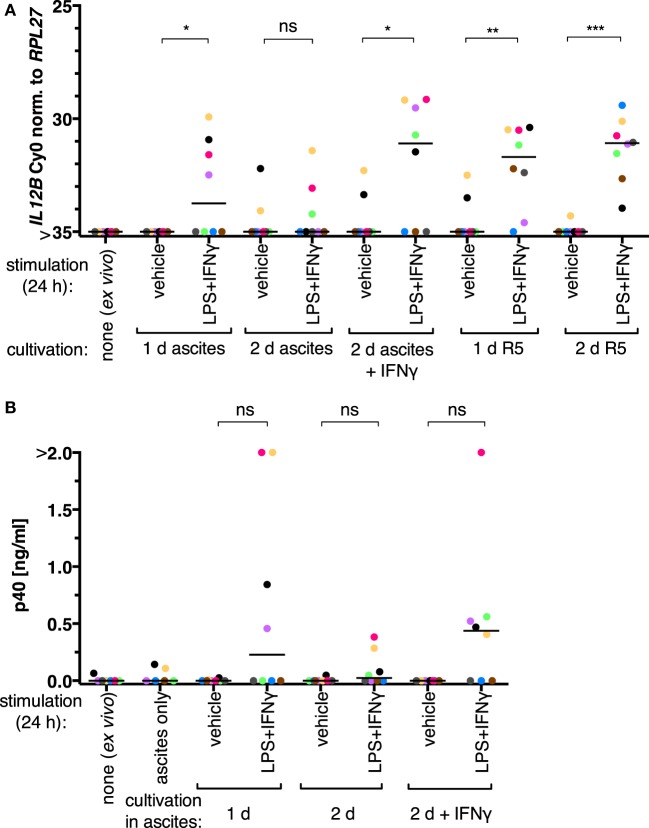
IL-12 production by tumor-associated macrophages from ovarian carcinoma patients is suppressed by ascites *in vitro*. **(A)** *Ex vivo* TAMs (*N* = 8) were cultivated in autologous ascites in the presence or in the absence of IFNγ or in normal medium (R5) as indicated, and non-cultivated *ex vivo* TAMs served as an additional control. LPS and IFNγ or their respective solvents (vehicle) were added to the culture supernatants for 24 h prior to harvesting, and *IL12B* expression was measured by RT-qPCR. **(B)** Secreted IL-12p40 from these TAMs was measured by ELISA (*N* ≥ 6). Cell-free autologous ascites was used as additional control. Each dot denotes a biological replicate. Median values are indicated by horizontal bars. Colors encode individual patients, and colors are consistent between panels within this figure. Statistical significances were calculated with paired *t*-tests. *P < 0.05; **P < 0.01; ***P < 0.001; ns, not significant.

We initially assessed whether the inability of TAMs to produce IL-12p40 is reversible in our experimental setups. *Ex vivo* TAMs were in autologous ascites or in normal medium (RPMI 1640 supplemented with 5% adult human serum, “R5”) for 1 day or 2 days and stimulated with LPS and IFNγ 24 h prior to harvesting. *IL12B* expression was induced on both mRNA (Figure 1A; RT-qPCR) and protein levels (Figure 1B; α-p40 ELISA) in cells from four out of eight patients cultivated in ascites. After cultivation in autologous ascites for 2 days, the transcript and the protein were inducible to a lesser extent; LPS and IFNγ were added to the cultures 24 h prior to harvesting. The presence of IFNγ for the whole time of cultivation in 2 days samples led to increased induction. We conclude that suppression of *IL12B* in TAMs happens primarily at the transcriptional level, and suppression can be counteracted by IFNγ, as it was shown by others (34). Furthermore, we found that expression was inducible without IFNγ pretreatment in some samples (Figure 1). It is important to note that we added IFNγ in parallel with LPS to non-pretreated cultures, while the aforementioned study used LPS as a single stimulus after pretreatment with IFNγ (34). Apparently either a subpopulation of the cells from these patients is responsive to LPS and IFNγ *ex vivo*, or the autologous ascites samples are less capable of *IL12B* suppression. Strikingly, TAMs cultivated in normal medium in the absence of IFNγ were able to induce *IL12* transcription to similar levels as TAMs cultivated in ascites in the presence of IFNγ (Figure 1A). Cultivation in normal medium led to detectable transcripts in all donors analyzed (*N* = 8), while cultivation in the presence of IFNγ had this effect only in five out of eight donors. Taken together, this suggests that ascites-mediated suppression of *IL12B* induction is reversible, and this may depend on differences between ascites samples as well as cells from individual donors. Presumably due to biological heterogeneity, differences between the induced and non-induced conditions regarding p40 protein levels were not statistically significant when analyzing supernatants from TAMs cultivated in ascites (Figure 1B).

*IL12B* expression data from TAMs cultivated in normal medium or in ascites supplemented with IFNγ suggest that these cells are generally capable of inducing this transcript. Its induction in the presence of ascites could be due to an effect on monocytic cells that were freshly recruited to the tumor microenvironment and thus not yet strongly affected by ascites, or the effect of ascites on *IL12B* could be of a reversible nature. We, therefore, conducted the following experiments in MDMs differentiated from healthy donor monocytes *in vitro* in normal medium (R5) or in ascites as described previously (7)—this culture system allows for controlled differentiation as well as short-term treatment of cell populations that are less heterogeneous than TAM isolates. High levels of IL-12p40 can be produced by MDMs, and this is suppressed by ascites but overcome in the presence of IFNγ (7, 34). Priming of MDMs differentiated in R5 with IFNγ prior to stimulation with LPS and IFNγ did not potentiate IL-12p40 production (Figure S1 in Supplementary Material), indicating that our culture conditions do not limit expression of p40 in this regard.

### 3.2 Suppression of *IL12B* Can Be Reversible Upon Ascites Withdrawal

**Figure 2:**
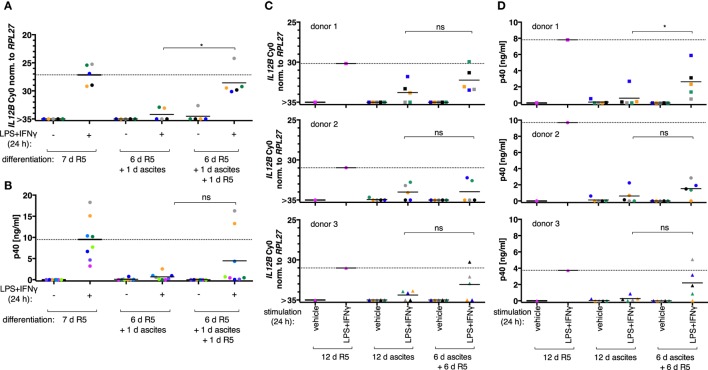
Ascites-mediated suppression of *IL12B* in monocyte-derived macrophages is reversible upon ascites withdrawal. *IL12B* expression induced by LPS and IFNγ in MDMs (differentiated as indicated) was measured by RT-qPCR **(A,C)** or in culture supernatants by ELISA **(B,D)**. Cells were stimulated for 24 h prior to harvesting, or the respective solvents (vehicle) were added, respectively. **(A,B)** Each dot denotes a biological replicate (healthy donor; *N* ≥ 5) randomly combined with different ascites samples encoded by color. **(C,D)** Colors encode ascites samples from five different patients; shapes encode different healthy donors (*N* = 3). Median values are indicated by horizontal bars. Statistical significances were calculated with paired *t*-tests. *P < 0.05; ns, not significant.

When MDM differentiation was carried out in normal medium for 6 days and followed by exposure to ascites for 1 day (short-term exposure, which was applied simultaneously with LPS and IFNγ), *IL12B* (Figure 2A, *N* = 5), and IL-12p40 (Figure 2B, *N* = 8) induction was efficiently suppressed. The effect was reverted in all MDM samples on RNA level by ascites withdrawal for 1 day after short-term exposure (Figure 2A), which was statistically significant; however, cells from most donors produced little or no p40, while others produced considerable levels (3/8; Figure 2B). This further point to a highly variable effect due to heterogeneity between biological samples, which could be due to the use of different ascites samples, different healthy donors, or both.

To clarify whether long-term exposure to ascites has lasting effects on the capacity of MDMs to express *IL12B*, monocytes from three healthy donors were differentiated either in R5 or in each of five different cell-free ascites samples from OC patients for 12 days (long-term exposure) in order to mimick the cellular state after differentiation in the ovarian tumor microenvironment. *IL12B* induction by LPS and IFNγ was markedly reduced in ascites-differentiated MDMs vs. MDMs differentiated in R5 medium on both mRNA (Figure 2C) and protein levels (Figure 2D). Although, suppression was not complete in some cultures, and this depended on both the donor cells and the ascites samples. MDMs differentiated in ascites for 6 days clearly induced *IL12B* expression after ascites withdrawal for 6 days (Figures 2C,D), albeit to lower levels than MDMs cultivated in normal medium for 12 days. Importantly, this happened in MDM cultures from all three donors in combinations with different ascites samples; p40 was detected in all but two combinations of donor cells with ascites samples. Induction after ascites withdrawal was elevated, but the effect did not reach statistical significance except for one comparison (Figure 2D, upper right panel). This can be attributed to the limiting number of samples analyzed and, importantly, to biological variation. Some ascites samples exerted suppressive effects that resulted in no or lesser reversibility, and MDMs from individual donors were susceptible to suppression by different ascites samples in a differential manner. We conclude that suppression of *IL12B* expression by OC ascites is reversible by ascites withdrawal in principle. On RNA level, reversal was more effective after short-term exposure. Since suppression was functional when ascites was added simultaneously with LPS and IFNγ, these data suggest that a suppressive mechanism acts immediately at the level of transcription and can affect MDMs which were differentiated in the absence of ascites (Figure 2A). Additional suppressive mechanisms apparently act posttranscriptionally (Figure 2B).

The observation that *IL12B* suppression by ascites is rapid and reversible suggests that at least some of the mechanisms involved do not elicit a stable macrophage polarization state. In order to systematically characterize the state of MDMs upon suppression of *IL12B* expression by ascites *in vitro*, monocytes were differentiated in normal medium, in ascites in the presence of recombinant IFNγ, or in normal medium for 6 days followed by ascites for 1 day (short-term exposure). Under these conditions, we found that ascites leads to increased expression of the markers CD163 and CD206, indicating alternative polarization (Figure S3 in Supplementary Material). Upon cultivation in ascites in the presence of IFNγ, induction of these markers was partially reversed, concomitant with a restoration of *IL12B* inducibility. It can, therefore, not be excluded that an altered macrophage differentiation state might contribute to the blockade of *IL12B* transcription in TAMs. This notion would be consistent with the observed stable *IL12B* suppression by a prolonged exposure of MDMs to ascites.

### 3.3 Ascites Reversibly Suppresses Nuclear Translocation of REL and p65

Since suppression of *IL12B* induction by ascites is immediate and acts on the level of transcription, an obvious assumption is that NFκB function is compromised due to high levels of IL-10 in ascites. Nuclear translocation of REL was detected after both 1 and 2.5 h of stimulation with LPS and IFNγ in our MDM culture system in cells differentiated in normal medium (Figure S2B in Supplementary Material), and the latter time point coincided with measurable synthesis of *IL12B* mRNA (Figure S2A in Supplementary Material). This is in line with the regulation of “second wave” NFκB target genes such as *IL12B* (51). For subsequent analyses, LPS and IFNγ were added to the culture supernatants 2.5 h prior to harvesting.

**Figure 3:**
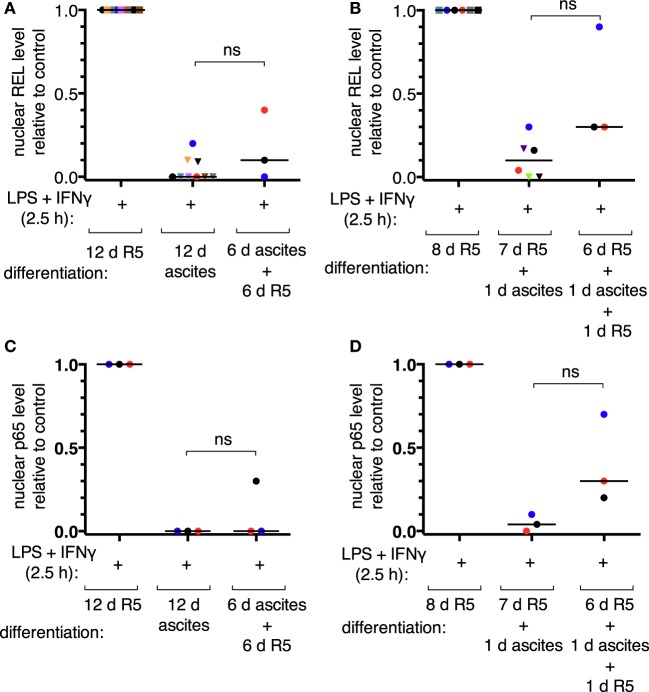
Ascites impairs inducible nuclear translocation of REL and p65 in monocyte-derived macrophages. MDMs were differentiated in R5 medium or in ascites and cultivated consecutively as indicated. Cells were incubated with LPS and IFNγ for 2.5 h or their respective solvents (vehicle), harvested, and subcellular fractionation was performed. After immunoblotting, chemoluminescence was measured, nuclear levels of REL were calculated relative to the R5 control population after long-term ascites exposure with (*N* = 9) or without (*N* = 3) ascites withdrawal **(A)** or after short-term exposure **(B)**, respectively (*N* = 6 or *N* = 3 as plotted). Nuclear p65 levels were analyzed in a subset of the same samples (*N* = 3) accordingly **(C,D)**. Each color denotes a biological replicate (combination of healthy donor and randomly chosen ascites sample). Dots mark samples which were probed for both REL and p65 nuclear translocation. The code is consistent between panels within this figure. Median values are indicated by horizontal bars. Statistical significances were calculated with paired *t*-tests; ns, not significant (*P* > 0.05).

According to our mass spectrometry data, REL, p65, and p50 are the main NFκB transcription factors expressed in TAMs (52). Nuclear translocation of the transcriptional activators REL and p65 was assessed by subcellular fractionation of MDMs after short-term (24 h) or long-term exposure to ascites (differentiation for 6 days or more). Representative immunoblots are shown in Figures S4B,C in Supplementary Material. Long-term exposure abrogated measurable REL translocation in six out of nine donors (Figure 3A) and translocation of p65 in all three donors analyzed (Figure 3C). Short-term exposure was less effective; detectable REL translocation was lost in two out of six donors (Figure 3B) and that of p65 in one out of three (Figure 3D). Although, exposure to ascites strongly reduced nuclear translocation in cells from all donors. Strikingly, ascites withdrawal for 1 day reinstalled nuclear localization of both REL (Figure 3B) and p65 (Figure 3D) in MDMs exposed to ascites for 24 h, which, however, did not reach the same levels as those of MDMs not exposed to ascites. In cells from the same donors used for short-term exposure experiments, reinstallation happened only in two out of three donors for REL (Figure 3A) and in one out of three donors for p65 (Figure 3C) after long-term exposure and 6 days of ascites withdrawal. Taken together, this demonstrates that impairment of REL and p65 nuclear translocation by ascites is rapid, and the effect can be reversible upon ascites withdrawal.

Nuclear localization of the NFκB transcriptional activators is controlled by IκB proteins, and their involvement in IL-10-mediated impairment of NFκB function is well documented (46, 53). We, therefore, measured the levels of IκBα, Iκβα, and IκBε proteins after stimulation of MDMs with LPS and IFNγ (Figures S5A–C in Supplementary Material; representative immunoblots are shown in Figure S4A in Supplementary Material) and found that, despite nuclear localization of REL, cellular IκBα levels were increased upon stimulation in all cell populations analyzed (Figure S5A in Supplementary Material). The levels of IκBβ and IκBε were strongly decreased upon stimulation under all conditions (Figures S5B,C in Supplementary Material). This is in line with the described positive feedback mechanism of IκBα after its initial rapid degradation, which subsequently leads to enhanced protein levels (53). Inducible Iκβα degradation was more pronounced in cells differentiated in ascites (Figure S5B in Supplementary Material). In conclusion, we did not observe a correlation between the levels of IκB proteins and the diminished capacity of *IL12B* transcription in MDMs exposed to ascites relative to non-exposed cells.

### 3.4 Induction of *CXCL10* Expression Is Not Prevented in the Presence of Ascites

Because nuclear translocation of REL and p65 is diminished in the presence of ascites, we speculated that transcription of target genes other than *IL12B* may be affected. *CXCL10*/*IP10* is a *bona fide* REL (54–56) and p65 target gene (57) which, however, is expressed in TAMs (7). A subset of cDNA samples shown in Figure 2 was used to test ascites-mediated effects on the induction of *CXCL10* expression. We found that the levels of this highly inducible transcript—ΔCy0 ≥ 10 in most sample combinations, which is equivalent to >1,000-fold induction—were weakly reduced after short-term exposure, reaching statistical significance, and significantly elevated after short-term exposure and ascites withdrawal (Figure S6A in Supplementary Material). However, mean *CXCL10* levels (calculated for a total of six samples from three individual donors) were unaffected in MDMs stimulated with LPS and IFNγ after long-term exposure and ascites withdrawal (Figure S6B in Supplementary Material). *CXCL10* expression was uniformly high upon induction after both short-term and long-term exposure to ascites (Figures S6A,B in Supplementary Material). In some MDM populations, transcript levels were inducible to even higher levels compared to the control sample after long-term exposure (Figure S6B in Supplementary Material). In summary, this argues against a direct effect of ascites on *CXCL10* transcription. Therefore, our data do not show that mRNA synthesis of *CXCL10* is prevented in the presence of ascites. This argues against a general ascites-dependent perturbation of the function of REL and p65 provided that *CXCL10* is a direct target gene.

### 3.5 Chromatin Marks and Regulatory Elements at the *IL12B* Locus

**Figure 4:**
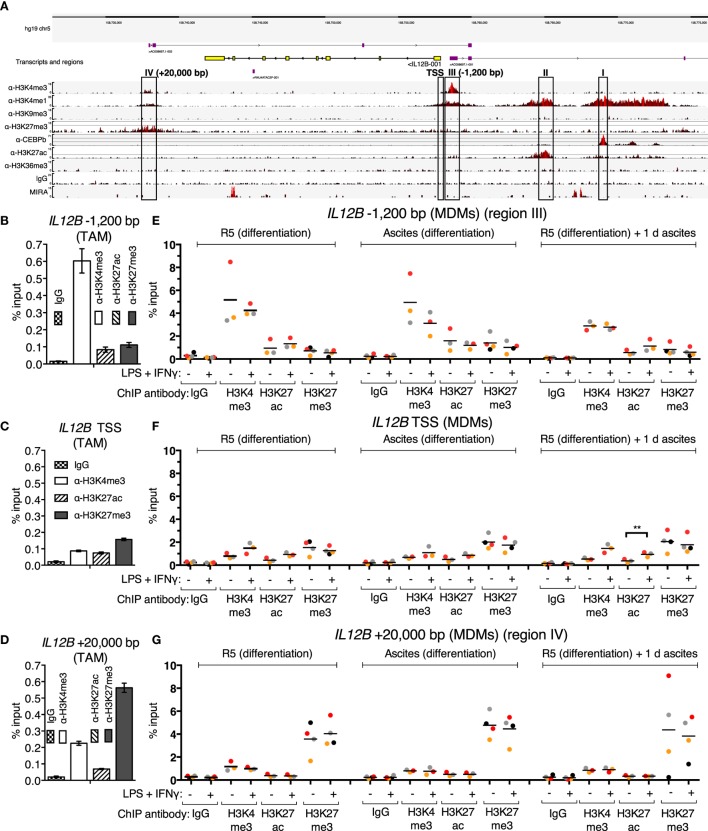
Chromatin marks at the *IL12B* locus. **(A)** A genome browser snapshot, including ChIP-seq tracks for α-H3K4me3, α-H3K4me1, α-H3K9me3, α-H3K27me3, α-C/EBPβ, α-H3K27ac, α-H3K36me3, IgG (unspecific polyclonal rabbit IgG pool) chromatin immunoprecipitations, and MIRA from a TAM sample freshly isolated from ovarian carcinoma ascites. Regions of interest are highlighted by rectangles. **(B–D)** ChIP-qPCR analyses of the indicated histone marks in an independent TAM sample at the indicated genomic locations amplified by specific primers are indicated. The error bars denote SDs from technical PCR replicates. **(E–G)** ChIP-qPCR analyses of the indicated histone marks in MDMs from three different pooled donor populations (*N* = 3; *N* = 4 for IgG and α-H3K27me3 samples) differentiated in normal medium, in ascites, or in normal medium followed by ascites for 1 day. Cells were stimulated with or without LPS and IFNγ (+) or their respective solvents (−) 2.5 h prior to harvesting. Genomic regions were amplified by specific primers are indicated. Each dot denotes a biological replicate; for each replicate, MDMs from six donors were pooled after harvesting of the cells for each experiment. Median values are indicated by horizontal bars. Colors encode ascites samples from individual patients, and colors are consistent between panels within this figure, Figure 5 and Figure S9 in Supplementary Material. Statistical significances were calculated with paired *t*-tests. **P < 0.01; all other analyses did not show significance (*P* > 0.05).

Since translocation of REL and p65 is impaired in the presence of ascites, and *IL12B* mRNA induction is prevented, while that of *CXCL10* is not, an obvious hypothesis is that these transcripts are subject to gene-specific regulation. In order to address ascites-mediated effects on NFκB target genes, which are possibly locus-specific, we sought to map histone modifications and regulatory elements at these REL and p65 target genes in primary macrophages in an unbiased approach. To this end, ChIP-seq was performed in *ex vivo* OC TAMs with antibodies against the histone modification marks H3K4me1 (histone H3 lysine 4 monomethylation), H3K4me3, H3K9me3, H3K27me3, H3K27ac (H3K27 acetylation), H3K36me3, as well as the transcription factor C/EBPβ. Genome browser snapshots of the *IL12B* locus (Figure 4A) indicate that H3K4me1, which marks enhancer sequences, was detected at four sequence stretches within 25 kbp from either end of the coding region. These stretches are distinguished by the following features: (I) C/EBPβ binds to a region 11 kbp upstream of the *IL12B* transcription start site (TSS); (II) a region 7 kbp upstream of the TSS is decorated with H3K27ac, which marks active positive regulatory elements; (III) a local enrichment of H3K4me3 is localized 1,200 bp upstream of but not at the TSS itself, where this mark is usually found at active and poised genes (58); (IV) a region 4,500 bp downstream of the gene (20 kbp downstream of the TSS) harbors H3K27me3-modified nucleosomes, indicative of the Polycomb repressive complex 2 (58).

All four designated regions harbor highly conserved sequences, and none of them carry the heterochromatin mark H3K9me3. Consistent with minute or absent expression of *IL12B* transcripts in *ex vivo* OC TAMs (7), the coding region is devoid of the transcription elongation marker H3K36me3. Poorly annotated transcripts originate from regions III and IV. These long non-coding RNAs generally correlate with regulatory roles of the respective DNA sequences (59). Region II possibly is in spatial proximity to region I, since a minor but noticeable enrichment was caused by the C/EBPβ antibody at this site, which could be due to indirect crosslinking. We assume that regions I and II do not mediate repression due to the presence of the activating H3K27ac mark at region II and the absence of repressive marks at both regions.

The unusual configuration of the H3K4me3 mark appearing at a −1,200 bp upstream site (region III) but not at the TSS seems to be a hitherto undescribed characteristic of a set of cytokine-encoding genes: in our dataset, we observed similar distances at, for instance, *CXCL10/IP10* (−5,000 bp) and *IL2* (−2,000 bp), which both are REL target genes (42, 54–56, 60). Genome browser snapshots of both loci are shown in Figures S9A,B in Supplementary Material. *CXCL10* is expressed in OC TAMs as well as in peritoneal macrophages from tumor-free patients (6, 7), while *IL2* is not a transcribed gene in macrophages and hence is devoid of active marks but apparently harbors H3K4me3- and H3K4me1-modified nucleosomes within 2,000 bp of its TSS. We excluded the possibility that these H3K4me3 upstream shifts relative to the TSSs are due to mapping artifacts; moreover, these observations were confirmed in published datasets such as those from ENCODE (ChIP-seq tracks for H3K4me3; GSM1003536 and GSM945225 for monocytes; and GSM788075 for PBMCs) and an early ChIP-seq study in the Jurkat human T cell line (61).

Strikingly, a weak enrichment of region IV was also caused by the H3K4me3 antibody, which strongly enriches region III. Reciprocally, reads from the α-H3K27me3 ChIP, which are most prominent at region IV, are more densely spaced in an extended stretch, which encompasses region III and the TSS of *IL12B*, relative to the coding region. Taken together, region IV may be in proximity to region III in OC TAMs, since each region’s more prominent of these two histone marks is, to a lesser extent, mirrored at the other region, presumably due to indirect crosslinking. The presence of both H3K4me3 and H3K27me3 at the same locus is reminiscent of the bivalent state which allows for stable repression and comparably fast induction of expression that was first described for developmental genes (58); however, bivalent genes carry the marks at or close to their TSSs.

To investigate whether cytosine methylation is involved in repression of *IL12B* in TAMs, we used MIRA (methylated CpG island recovery assay)-seq (62). CpG island methylation was not detected at the promoter region of *IL12B*, while robust signals originated at the penultimate exon and a region 9 kbp upstream of the TSS (Figure 4A). Importantly, the promoter as well as conserved sequences upstream (region I) and downstream of the coding region, 20 kbp from the TSS (region IV), harbor CpG islands according to DBCAT analysis (63), but were not enriched by MIRA. The human Jurkat T cell line and other cell lines analyzed show an enrichment of methylated CpG sequences at the promoter and region IV according to ENCODE datasets (with GEO accession numbers; Jurkat: GSM999367, HeLa-S3: GSM999337, H1: GSM999379, HepG2: GSM999338, HL-60: GSM999386, GM12878: GSM999376, K562: GSM999341, HUVEC: GSM999364, Ovcar-3: GSM999393). This potentially means that methylation of region IV and the promoter regulates cell-type specific expression of *IL12B*.

In summary, the next-generation sequencing analyses led to the hypothesis that suppression of *IL12B* in TAMs is mediated by an H3K27me3-dependent mechanism and involves the promoter/−1,200 bp region and a putative silencer element 20 kbp downstream of the TSS. For further analyses, we focused on elements that harbor repressive marks (regions III: −1,200 bp and IV: +20 kbp) as well as the TSS. The observed enrichment of H3K4me3 and H3K27me3 at these elements was confirmed by ChIP-qPCR in a different TAM sample (Figures 4B–D).

Using ChIP-qPCR, analysis of the chromatin marks H3K4me3, H3K27ac, and H3K27me3 was performed in MDMs differentiated in normal medium, in ascites, or in normal medium followed by exposure to ascites for one day in order to measure possible changes upon short-term and long-term suppression (Figures 4E–G). Cells were stimulated with LPS and IFNγ 2.5 h prior to fixation; at this time point, REL and p65 were detected in the nucleus (Figure 3; Figure S2B in Supplementary Material), and *IL12B* transcripts were detectable shortly thereafter (Figure S2A in Supplementary Material). The observed relative levels of the analyzed histone modifications generally mirrored the observations made in TAMs (Figures 4B–D), with absolute recoveries in MDMs being higher due to lower cell numbers that were obtained from MDM cultures in comparison to TAMs from large volumes of ascites. H3 lysine 4 trimethylation levels did not change consistently; at the TSS, levels were low but uniformly increased upon stimulation with LPS and IFNγ. At the *IL12B* TSS, the H3 lysine 27 acetylation signal increased consistently (Figure 4F) upon stimulation, and this was statistically significant in the short-term ascites-exposed population. Mean H3K27me3 signals at the TSS of *IL12B* were slightly elevated in MDMs exposed to ascites. The difference did not reach statistical significance. Upon stimulation, α-H3K27me3 signals were slightly diminished at the *IL12B* TSS (Figure 4F). This might reflect a decreased interaction between the TSS and the downstream putative silencer element at +20,000 bp, where robust enrichment of H3K27me3 was detected (Figure 4G). In summary, it seems plausible that the region IV downstream site and the TSS/−1,200 bp regions are in spatial proximity to each other, and H3K27me3 at the TSS might possibly be elevated after exposure to ascites. However, we cannot rule out that H3K27me3-dependent mechanisms are dispensable for suppression of *IL12B* due to the observation that enrichments by α-H3K27me3 at the TSS was only mildly elevated in MDMs exposed to ascites relative to the control population. The identified putative regulatory elements at the *IL12B* and *CXCL10* loci are candidate regions for assessing binding of transcription factors that regulate *IL12B* expression.

### 3.6 Inducible REL and p65 Binding to Chromatin Is Not Impaired in MDMs Exposed to Ascites

**Figure 5:**
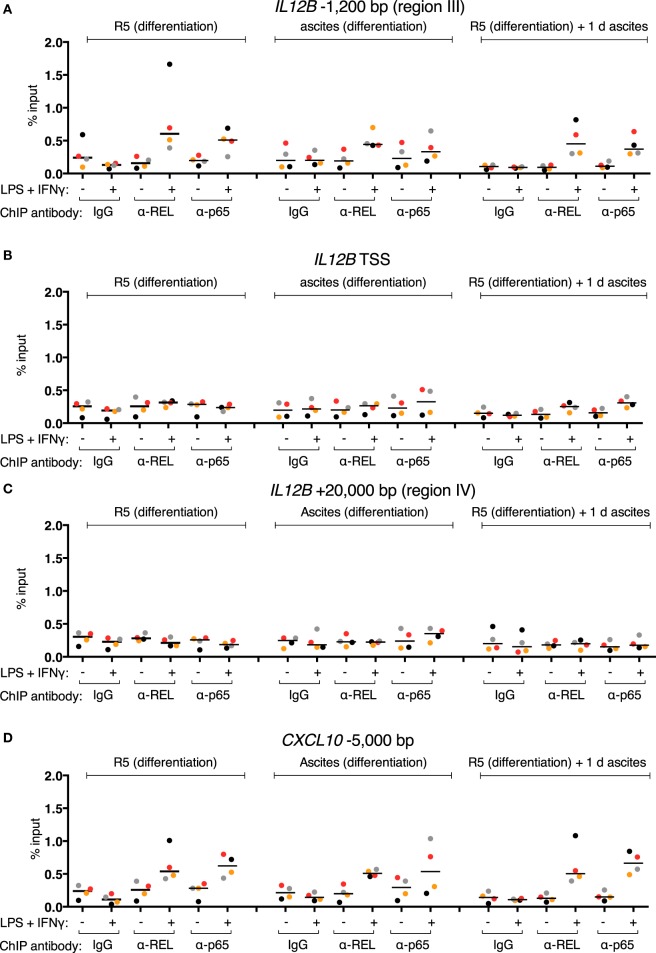

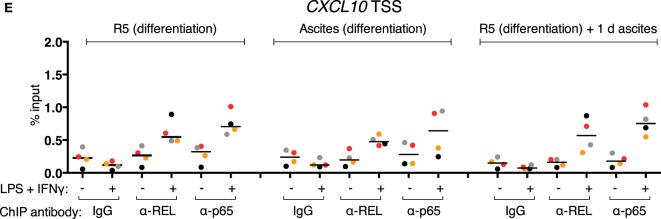
Inducible REL and p65 binding at the *IL12B* and *CXCL10* loci in MDMs. **(A–E)** ChIP-qPCR analyses of REL and p65 binding in chromatin preparations from MDMs differentiated in normal medium, in ascites, or in normal medium followed by ascites for 1 day were conducted. Cells were stimulated with or without LPS and IFNγ (+) or their respective solvents (−) 2.5 h prior to harvesting. Genomic regions were amplified by specific primers are indicated. The same samples were used as in Figure 4 (*N* = 4). Each dot denotes a biological replicate; for each replicate, MDMs from six donors were pooled after harvesting of the cells. Median values are indicated by horizontal bars. Colors encode ascites samples from individual patients, and colors are consistent between panels within this figure, Figure 4 and Figure S9 in Supplementary Material.

REL and p65 binding was measured (Figures 5A–C) in the same samples as in Figures 4E–G (identical data for IgG samples are shown). An enrichment with antibodies against each transcription factor was induced in the control population after stimulation at *IL12B* −1,200 bp (Figure 5A). Surprisingly, in the short-term ascites exposed population, REL and p65 were recruited to a similar extent at the −1,200 bp site of *IL12B*, while long-term exposure to ascites led to reduced but measurable recruitment. No recruitment was observed at the TSS and at +20,000 bp (Figures 5B,C). This indicates that chromatin binding of the NFκB transcriptional activators is functional in the presence of ascites despite their strongly impaired translocation (Figure 3). Notably, at the *CXCL10* locus, where REL and p65 recruitment was detected at both the TSS (Figure 5D) and an upstream element (Figure 5E), recruitment was enhanced by short-term ascites exposure but was unchanged after long-term exposure. These data show that, unexpectedly, nuclear REL and p65 levels are not measurably limiting for their binding to chromatin in MDMs exposed to ascites.

### 3.7 The Role of REL in the Induction of *IL12B* Expression

**Figure 6:**
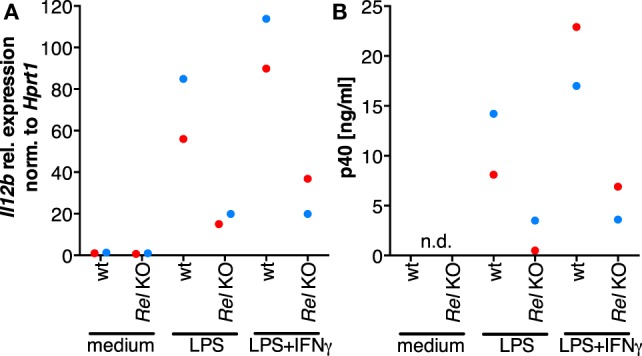
The role of Rel in the induction of *Il12b* expression. Murine *Rel* knockout BMDMs or wild-type BMDMs (*N* = 2 each) were differentiated *in vitro* and stimulated with LPS or LPS and IFNγ for 24 h. Cells were used for RT-qPCR analysis of *Il12b* **(A)**, and Il-12p40 was measured in supernatants by ELISA **(B)**; n.d., not detected.

In order to clarify whether RNAi-mediated depletion of REL and p65 affects expression of *IL12B*, we employed knockdown approaches using siRNA in MDMs differentiated in normal medium. Functionality of the siRNA oligonucleotides was validated on protein level (Figure S8 in Supplementary Material). However, we were unable to achieve high knockdown efficiencies in primary macrophages, resulting in apparent protein levels of 60% relative to the control population or less. Knockdown of REL resulted in slightly diminished induction of IL-12p40 (Figure S7 in Supplementary Material). In a murine *Rel* knockout model, induction was largely dependent on Rel (42). However, the effects we observed after RNA interference are mild, which we attribute to incomplete depletion of the target proteins and to the effect that transfection with control siRNA also led to a strong reduction of p40 expression in most experiments. We could not find an efficient transfection reagent that did not elicit this effect, even in the absence of siRNA oligonucleotides (data not shown). In one of the MDM populations analyzed, *IL12B* expression was affected neither by REL knockdown nor by p65 knockdown (blue dots, Figure S7 in Supplementary Material) despite a measurable reduction of their protein levels, and in another sample, induction was increased after knockdown of p65 (black dots, Figure S7 in Supplementary Material). This prompted us to use a different experimental system in which Rel is genetically deleted. When we stimulated murine *Rel* knockout bone marrow-derived MDMs (BMDMs) with LPS and IFNγ, induction of *Il12b* (Figure 6A) as well as p40 (Figure 6B) was reduced to about 20% of the levels generated by wild-type cells, as it was similarly shown by others previously. These data suggest that REL is important but not necessary for *IL12B* induction. Indeed, it was observed previously by others that Rel-deficient murine antigen-presenting cells can produce IL-12p40 to a highly varying extent depending on the tissue they were isolated from Ref. (64). The function of REL is conceivably supplemented by and partially redundant with that of p65, as it was noted before (42, 65). The mild effects we observed after siRNA-mediated partial depletion are in line with the notion that levels of REL and p65 are not limiting for target gene induction in MDMs.

### 3.8 IL-10 Is Not Sufficient to Suppress *Il12b* Expression

**Figure 7:**
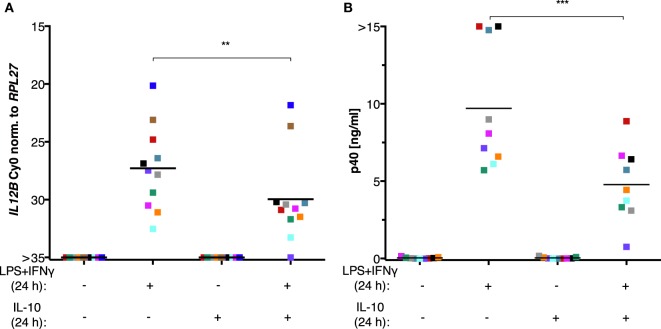
IL-10 is not sufficient to suppress *IL12B* induction in MDMs. MDMs were differentiated in R5 medium for 6–7 days and stimulated with LPS and IFNγ, with recombinant IL-10 from human cells, or both for 24 h. **(A)** *IL12B* expression was measured by RT-qPCR (*N* = 11). **(B)** IL-12p40 in culture supernatants (*N* = 9) was measured by ELISA. Each square denotes a biological replicate (individual healthy donor). Colors encode individual healthy donors, and colors are consistent between panels within this figure. Median values are indicated by horizontal bars. Statistical significances were calculated with paired *t*-tests. **P < 0.01; ***P < 0.001.

The inability of MDMs to synthesize IL-12p40 after exposure to ascites could be explained by the negative effect of IL-10 on the translocation of REL and p65 (45–47) as well as its indirect actions *via* the induction of STAT3 target genes (23, 24, 66–68). However, treatment of MDMs (differentiated in normal medium) with recombinant IL-10 from human cells in parallel with LPS and IFNγ did not recapitulate full suppression of IL-12p40 production (Figure 7B) and was unable to shut off *IL12B* transcription (Figure 7A) in cells from all but one donor. Importantly, the effects of partial suppression were highly significant on both mRNA (Figure 7A) and protein levels (Figure 7B), indicating that the recombinant IL-10 was functional. MDMs from some individual donors were affected more than others, demonstrating that the amplitude of suppression is donor-dependent to a large extent. We conclude that other ascites-borne factors are necessary for full suppression of *IL12B*.

## 4 Discussion

Our data suggest that (1) suppression of *IL12B* transcription by ovarian carcinoma ascites acts immediately and on differentiated macrophages, and it is reversible upon ascites withdrawal; (2) although ascites impinges on REL and p65 nuclear translocation, binding of these factors to chromatin is not diminished; and (3) IL-10 can only partially recapitulate suppression. This implicates that soluble factors in ascites may act combinatorially to achieve rapid, gene-specific suppression of *IL12B* transcription.

### 4.1 Reversibility and Immediacy of Ascites-Mediated *IL12B* Suppression

Immediately after ascites exposure, *IL12B* transcription was largely abrogated in MDMs simultaneously stimulated with LPS and IFNγ (Figure 2A). *IL12B* mRNA inducibility was partially restored in cells from all donors after ascites withdrawal. These data indicate that, while suppression is rapid, its effects are not permanent in cells exposed to ascites for 24 h. Additional posttranscriptional mechanisms negatively affecting p40 synthesis are likely (Figure 2B). Importantly, some MDM cultures retain the ability to secrete measurable amounts of p40 in the presence of ascites, and this apparently depends on both the donor and the ascites used. Ascites withdrawal further attenuates suppression (Figures 2A–D).

In line with reversibility of *IL12B* suppression, the transcript was detected in most *ex vivo* TAM samples in the absence of ascites (Figure 1A). *IL12B* expression reached levels similar to those in TAMs cultivated in ascites in the presence of IFNγ. Furthermore, exposure to ascites does not immediately induce macrophage M2 marker expression in MDMs (Figure S3 in Supplementary Material), an observation which is compatible with a model of transient suppression by ascites.

As mentioned before, our data indicate that biological variation is high among both donor cells and ascites samples, which agrees with our finding that, on transcriptome level, TAMs from OC patients can be clustered into two groups which differ in the expression levels of interferon-responsive genes. Higher expression of these genes is positively correlated with patient survival (7). From a therapeutical perspective, it might be beneficial to determine genetic predispositions and environmental factors involved in ascites-mediated suppression of *IL12B*.

### 4.2 Restrainment of NFκB by Ascites Is Not Complete

Ascites-exposed MDMs show strongly reduced REL and p65 nuclear translocation (Figure 3), yet chromatin binding of these factors is functional (Figure 5). Additionally, after short-term ascites exposure, REL and p65 translocation was not fully impaired in most samples (Figures 3B,D). In conclusion, nuclear REL and p65 levels after stimulus-dependent translocation are sufficient for saturation of their binding sites at the *IL12B* and *CXCL10* loci regardless of ascites-mediated reduction of nuclear translocation. Consistent with this, siRNA-mediated partial depletion of REL or p65 did not significantly influence *IL12B* induction (Figure 6A). However, it cannot be excluded that reduced binding of REL proteins to sites with lower affinity than at the *IL12B* and *CXCL10* loci has an indirect impact on *IL12B* transcription, e.g., by affecting the expression of a gene-specific coactivator.

Interestingly, IL-10 was reported to selectively inhibit expression of a subset of LPS-inducible genes in murine macrophages in the absence of endogenous IL-10, and *Il12b* was among the most strongly downregulated transcripts (69). Gene-specific mechanisms offer a conclusive explanation why other NFκB targets such as *CXCL10* are not suppressed by ascites (Figure S6 in Supplementary Material). Unaltered occupancy of REL and p65 at both the *IL12B* and *CXCL10* loci in the presence of ascites (Figure 5) strongly suggests that these gene-specific mechanisms do not affect NFκB chromatin binding. Induction of *CXCL10* expression depends on IFNγ, its receptor and STAT1 (70, 71), and also on NFκB (72). These factors are likewise required for *IL12B* expression. Induction of *IL12B* by LPS and IFNγ is not functional in MDMs exposed to ascites (Figure 2), while induction of *CXCL10* is (Figure S6 in Supplementary Material). *CXCL10* is expressed in *ex vivo* TAMs, while *IL12B* is not (7). The two genes are presumably not regulated by differential NFκB and STAT1 recruitment to chromatin in the presence of ascites. Gene-specific posttranslational modifications of transcription factors, availability of coactivators, regulation of DNA looping involving the region IV putative silencer element, or a repressor which does not affect the marks we used to characterize the chromatin state of *IL12B* may be involved in suppression.

### 4.3 The Role of IL-10 in *IL12B* Suppression

A plethora of studies ascribe a major role to IL-10 in the maintenance of homeostasis, including the prevention of IL-12 production *via* multiple mechanisms (23, 24, 39, 43, 45–47, 67, 68, 73). Our observation that IL-10 does not fully recapitulate ascites-mediated suppression of *IL12B in vitro* in cultures from most donors suggests that additional suppressive factors might be lacking. In addition to IL-10, prostaglandin E2 (73), phosphatidylserine (74), adenosine (75, 76), lysophosphatidic acid (33), polyunsaturated fatty acids (77), and α-fetoprotein (78, 79) were described to downregulate IL-12 production. Potential targets of soluble factors could be upstream signaling components, transcription factors, or cofactors.

In murine alveolar macrophages, the E3 ubiquitin ligase Trim29 was found to negatively regulate the host response after bacterial infection. Trim29, which is exclusively expressed in alveolar macrophages, can induce degradation of the Ikk regulatory subunit (80). This highlights a paradigm for tissue-specific regulation of the pro-inflammatory response. Analogously, the expression of a regulator impinging on IKK function could conceivably be modulated by soluble factors from ascites. A corepressor which suppresses *Il12b* expression in murine macrophages is Smrt (81). The study elucidates that the closely related proteins Ncor and Smrt can differentially repress sets of target genes. *Il12b* was repressed by Smrt exclusively, and Smrt could be displaced from the locus by IFNγ treatment. It is conceivable that ascites-borne soluble factors stablize SMRT or another gene-specific corepressor at the *IL12B* locus, since the expression of *CXCL10* is not suppressed by ascites. Transcription factors described to repress *IL12B* expression such as Nfil3 in mice (24) or *c*-MAF (82) might contribute to the establishment of a locus-specific repressive complex.

Intriguingly, the levels of IL-10 as well as those of arachidonic acid in ascites are negatively correlated with patient survival according to our previous studies, and IL-10 and arachidonic acid levels are synergistically correlated with poor prognosis (21). This suggests that IL-10 alone is not sufficient to exert its full pro-tumorigenic effect *in vivo*. We postulate that other factors are required in addition to IL-10 for the suppression of a pro-inflammatory, anti-tumorigenic macrophage phenotype that includes expression of IL-12p40. Imbalances of this postulated interplay which are integrated into deregulated *IL12B* expression might be involved not only in tumorigenesis but could also contribute to diseases with an autoimmune component (26, 27). Future studies will investigate putative combinatorial suppressive mechanisms using a panel of purified factors.

## Data Availability

The ChIP-seq datasets generated for this study can be found in the ArrayExpress repository at https://www.ebi.ac.uk/arrayexpress/experiments/E-MTAB-6297.

## Ethics Statement

All patient samples were obtained in accordance with the recommendations of the Ethics Commission of the Department of Medicine, Philipps University of Marburg with written informed consent from all subjects. All subjects gave written informed consent in accordance with the Declaration of Helsinki. The protocol was approved by the Ethics Commission of the Department of Medicine, Philipps University of Marburg.

## Author Contributions

AU performed most experiments. FF applied all bioinformatical methods. TA performed TAM ChIPs, MIRA assays, and ChIP-seq library preparation. NH performed *IL12B* induction kinetics analysis. BJ and FN performed ELISA experiments. AN performed high-throughput sequencing. UW contributed clinical samples. SR prepared TAM samples. AV performed all experiments with murine cells. AU, FF, AV, SR, SM-B, RM, and TA conceived and designed the experiments. All authors analyzed data. RM and TA supervised the study. TA wrote the manuscript. AU, SM-B, and RM made major contributions to the writing of the manuscript.

## Conflict of Interest Statement

The authors declare that the research was conducted in the absence of any commercial or financial relationships that could be construed as a potential conflict of interest.

## Abbreviations

APC, antigen-presenting cell; BMDM, bone marrow-derived macrophage; CD, cluster of differentiation; C/EBP, CCAAT/enhancer binding protein; ChIP, chromatin immunoprecipitation; ENCODE, encyclopedia of DNA elements; GEO, gene expression omnibus; IFN, interferon; IKK, IκB kinase; IL, interleukin; IκB, inhibitor of κ light chain enhancer of activated B cells; JAK, Janus kinase; LPS, lipopolysaccharide; MDM, monocyte-derived macrophage; MFI, mean fluorescence intensity; MIRA, methylated CpG island recovery assay; MSMD, Mendelian susceptibility to mycobacterial disease; NFκB, nuclear factor κ light chain enhancer of activated B cells; NK, natural killer; OC, ovarian carcinoma; PBMC, peripheral blood mononuclear cell; pMDM, monocyte-derived macrophage from a patient; REL, reticuloendotheliosis; RPL27, ribosomal protein 27, large subunit; RPMI, Roswell Park Memorial Institute; RRID, research resource identifier; STAT, signal transducer and activator of transcription; TAM, tumor-associated macrophage; Th, T helper; TSS, transcription start site.
