# Supplementary material for: Chromatin Binding of c-REL and p65 Is Not Limiting for Macrophage IL12B Transcription During Immediate Suppression by Ovarian Carcinoma Ascites
Source: Front Immunol. 2018 Jun 27;9:1425. doi: 10.3389/fimmu.2018.01425 (PMC6030372; doi:10.3389/fimmu.2018.01425)
Supplement: Supplementary file 1 [file presentation_1.PDF]

frontiers

Supplementary Material: Chromatin binding of *c*-REL and p65 is not limiting for macrophage *IL12B* transcription during immediate suppression by ovarian carcinoma ascites

FIGURE CAPTIONS

1. Pretreatment with IFNγ does not lead to elevated p40 synthesis by MDMs cultivated in RPMI supplemented with adult human serum. MDMs were differentiated for 6–7 d in RPMI 1640 media supplemented with 5 % adult human AB serum (R5) and treated with IFNγ for the indicated periods of time prior to stimulation with LPS and IFNγ together or with vehicle for 24 h. IL-12p40 concentrations in culture supernatants were measured by ELISA. Two biological replicates denoted by colored symbols are shown.
2. Kinetics of *IL12B* induction and REL nuclear translocation in MDMs. MDMs were differentiated for 6–7 d in R5 media and incubated with vehicle or LPS and IFNγ for the indicated periods of time. **(A)** RT-qPCR was used to measure *IL12B* transcript levels (N=2). Dots represent mean values. **(B)** REL localisation was detected by immunoblotting after subcellular fractionation. LDH and acetylated histone H3 were used as both loading and fractionation controls. CE, cytoplasmic extract; NE, nuclear extract.
3. Macrophage M2 surface markers do not reflect immediate suppression of *IL12B* by ascites. MDM cultures (N=6) were differentiated in R5 media, in ascites, in ascites in the presence of 50 ng/ml recombinant for 7 d, or in R5 media for 6 d followed by ascites for 1 d. Cells were analysed by flow cytometry using α-CD163 **(A)** and α-CD206 **(B)** as probes. Data were plotted as percentage of positive cells or MFI (mean fluorescence index) as indicated. Each color denotes an individual donor, and donor MDMs were randomly combined with different ascites samples. Horizontal bars denote median values.
4. Degradation and translocation of NFκB pathway components (representative immunoblots). MDMs were differentiated in R5 media or in ascites and cultivated consecutively as indicated. Cells were incubated with vehicle or LPS and IFNγ for 2.5 h. **(A)** Whole-cell lysates were blotted, and membranes were probed with antibodies against IκB proteins as indicated. β-actin was used as a loading control. Relative calculated protein amounts are included in the panel. One representative blot is shown. **(B,C)** Cellular fractions were blotted, and membranes were probed with antibodies against REL or p65. LDH and acetylated histone H3 were used as both loading and fractionation controls. Relative calculated protein amounts are included in the panel. One representative blot is shown. CE, cytoplasmic extract; NE, nuclear extract.
5. Stimulus-dependent protein levels of IκBα, β and ε are not differentially affected upon exposure to ascites. **(A–C)** MDMs were differentiated in R5 media or in ascites and cultivated consecutively as indicated. Cells were incubated with or without LPS and IFNγ for 2.5 h, harvested and lysed. After immunoblotting of whole cell lysates, levels of IκB family members α (**A**, two biological replicates),

β (**B**, three biological replicates) and ε (**C**, three biological replicates) relative to the corresponding unstimulated sample were determined. Mean values are indicated by horizontal bars.

6. Induction of *CXCL10* expression is not prevented in the presence of ascites. *CXCL10* expression was measured by qRT-PCR in a subset of samples shown in fig. 2. MDMs were differentiated in R5 media or in ascites and cultivated consecutively as indicated. Stimulation was with LPS and IFNγ for 24 h prior to harvesting. Each symbol denotes a biological replicate (MDM donor). **(A)** Induction after differentiation in normal media, after short-term exposure to ascites, and after short-term exposure and withdrawal as indicated (N=4), samples correspond to fig. 2A. **(B)** Induction after differentiation in normal media, after long-term exposure to ascites, and after long-term exposure and withdrawal as indicated (N=3; cells from each donor were exposed to 1–3 different ascites samples as indicated; samples correspond to fig. 2C). Colors encode ascites samples from four (panel A) or three (panel B) different patients; shapes encode different healthy donors. The code is consistent with fig. 2A,C. Median values are indicated by horizontal bars. Statistical significances were calculated with paired t tests; *, $p<0.05$; ns, not significant.
7. MDMs differentiated in R5 media for 6–7 d (N=3) were transfected with siRNA as indicated, incubated for 24 h, stimulated with or without LPS and (+) or their respective solvents (vehicle; −) for an additional 24 h, and *IL12B* expression was measured by RT-qPCR. Black dots, Viromer transfection reagent; blue and green dots, Trans-IT X2 transfection reagent. Median values are indicated by horizontal bars. Each dot denotes a biological replicate. Statistical significances were calculated with paired t tests; ns, not significant ($p>0.05$).
8. Functionality of siRNA sequences on protein level (representative immunoblots). MDMs were differentiated for 6–7 d in R5 media. Cells were transfected with siRNA as indicated and incubated for 48 h. After immunoblotting of whole cell lysates, relative REL and p65 levels were calculated using LDH as a loading control. Relative calculated protein amounts are included in the panel. One representative blot is shown.
9. Chromatin marks at the *IL2* and *CXCL10* loci in TAMs and MDMs. **(A,B)** Genome browser snapshots including ChIP-seq tracks for α-H3K4me3, α-H3K4me1, α-H3K9me3, α-H3K27me3, α-C/EBPβ, α-H3K27ac, α-H3K36me3, IgG (unspecific polyclonal rabbit IgG pool) and MIRA from a TAM sample freshly isolated from ovarian carcinoma ascites encompassing the *IL2* (**A**) and *CXCL10* (**B**) genes. Regions of interest are highlighted with rectangles. **(C,D)** ChIP-qPCR analyses of the indicated histone marks in MDMs differentiated in normal media, in ascites, or in normal media followed by ascites for 1 d (the same samples as in figs. 6 and 7 are shown). Primers for the *CXCL10* 5000 bp upstream region (**C**) or the *CXCL10* TSS (**D**) were used, respectively. Each dot denotes a biological replicate (N≥3); for each replicate, MDMs from six donors were pooled after harvesting of the cells. Median values are indicated by horizontal bars. Colors encode ascites samples from individual patients, and colors are consistent between panels within this figure and figs. 6 and 7.
10. Original immunoblot images used for compilation of fig. 3. Please note that some of the blots are also shown in fig. S4. MDMs were differentiated in R5 media or in ascites and cultivated consecutively as indicated. Cells were incubated with vehicle or LPS and IFNγ for 2.5 h. Cellular fractions were blotted, and membranes were probed with antibodies against REL or p65. LDH and acetylated histone H3 or lamin B were used as both loading and fractionation controls. Relative calculated protein amounts are included in the panel. CE, cytoplasmic extract; NE, nuclear extract.

0.1 Figures

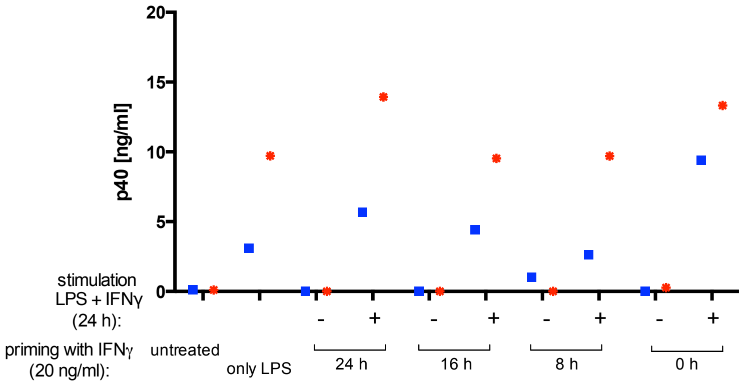

Figure S1: Pretreatment with IFNγ does not lead to elevated p40 synthesis by MDMs cultivated in RPMI supplemented with adult human serum.

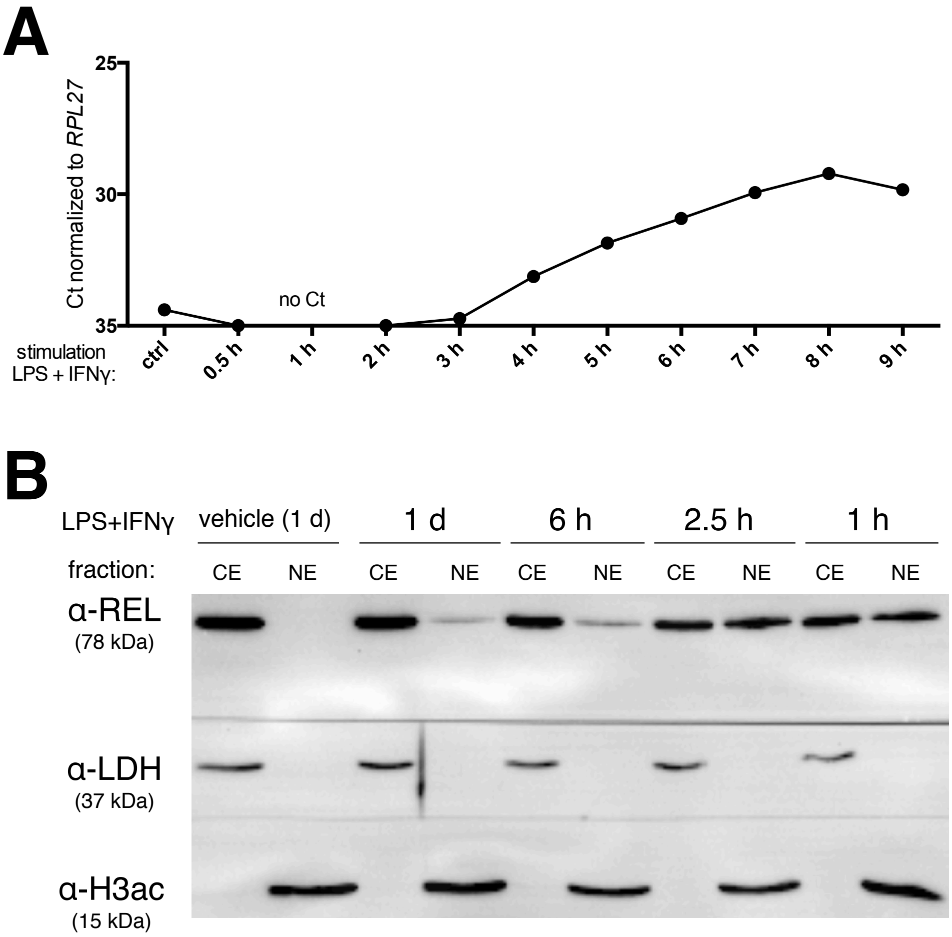

Figure S2: Kinetics of *IL12B* induction and REL nuclear translocation in MDMs.

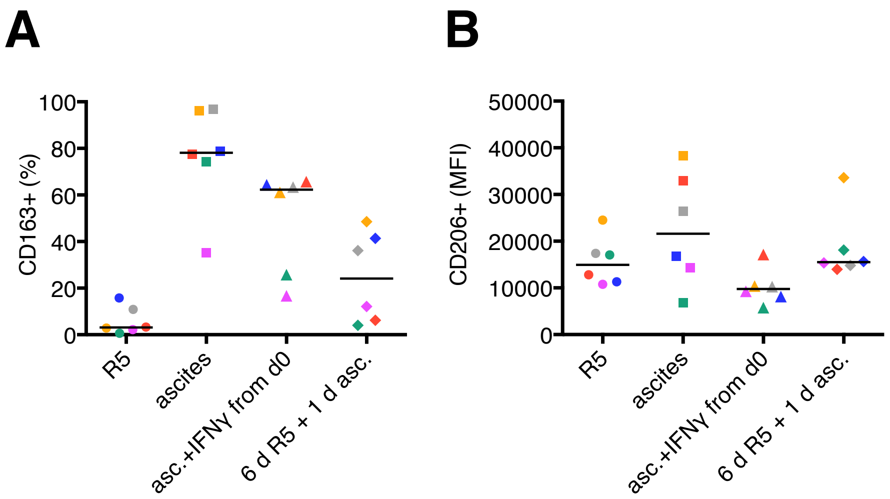

Figure S3: Macrophage M2 surface markers do not reflect immediate suppression of *IL12B* by ascites.

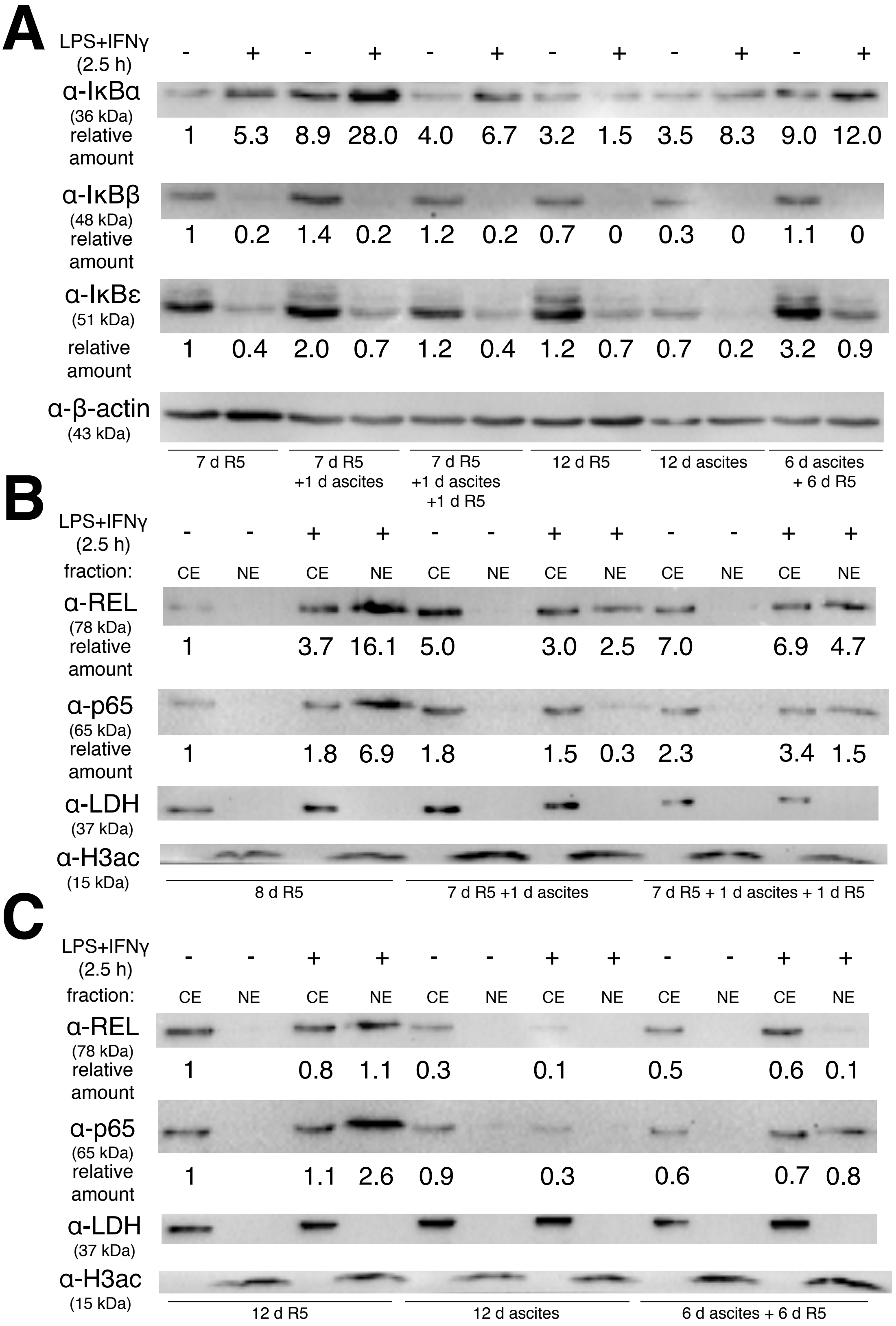

Figure S4: Degradation and translocation of NFκB pathway components (representative immunoblots).

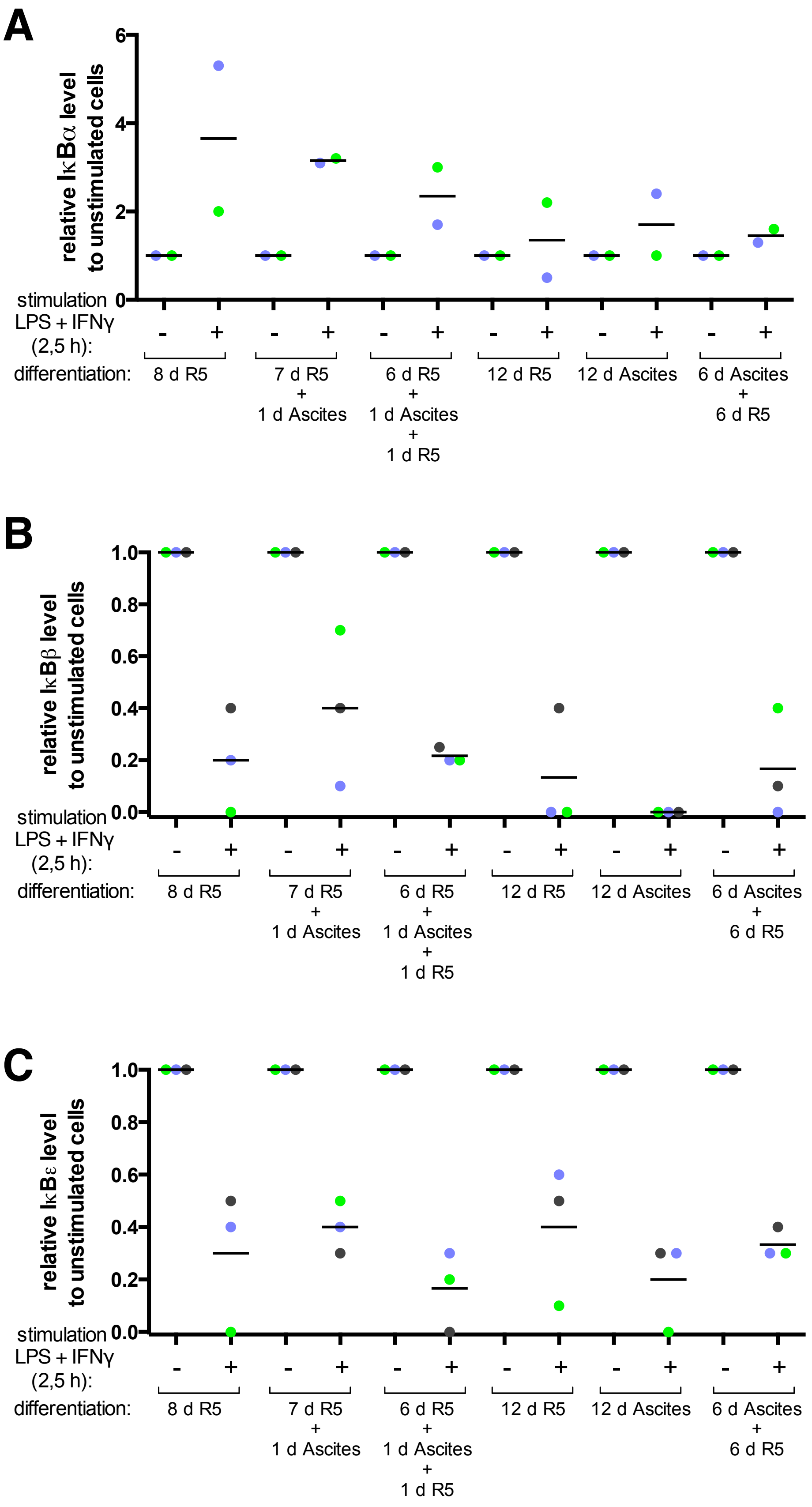

Figure S5: Stimulus-dependent protein levels of IκBα, β and ε are not differentially affected upon exposure to ascites.

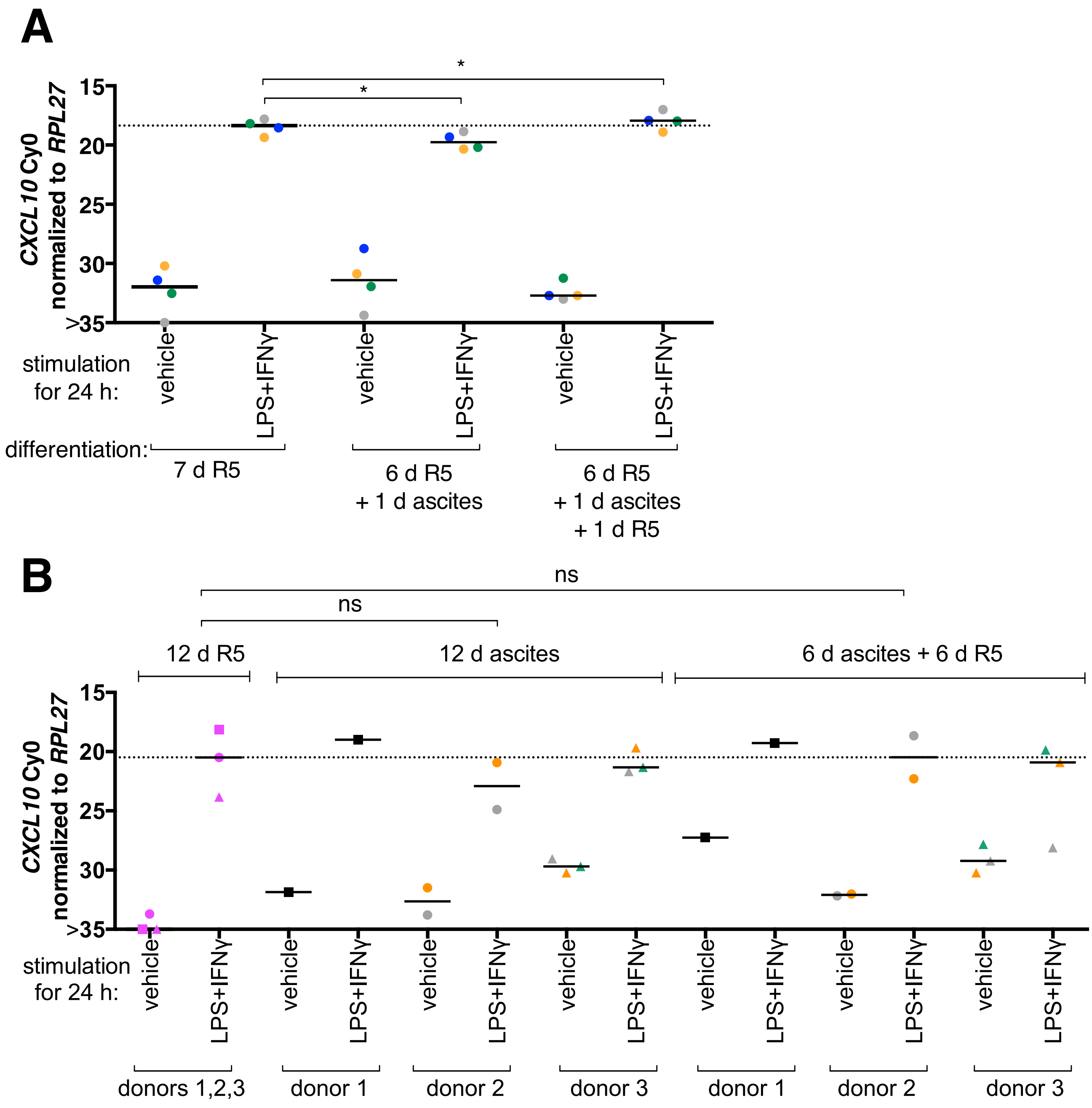

Figure S6: Induction of *CXCL10* expression is not prevented in the presence of ascites.

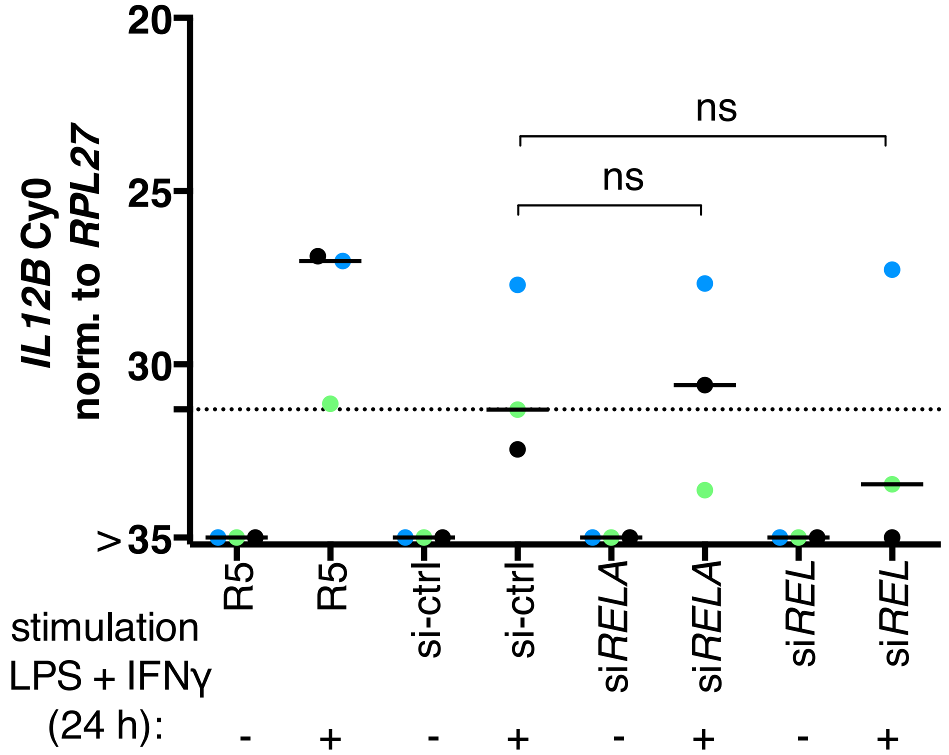

Figure S7: Induction of IL-12p40 expression after *REL* knockdown.

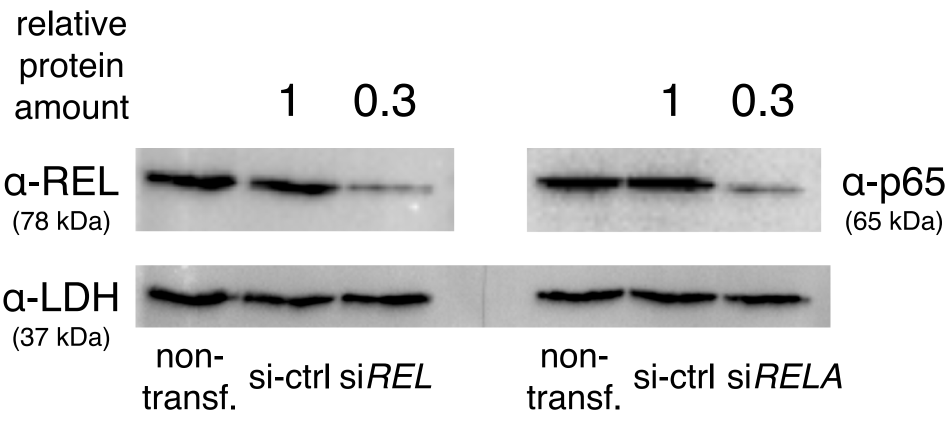

Figure S8: Functionality of siRNA sequences on protein level (representative immunoblots).

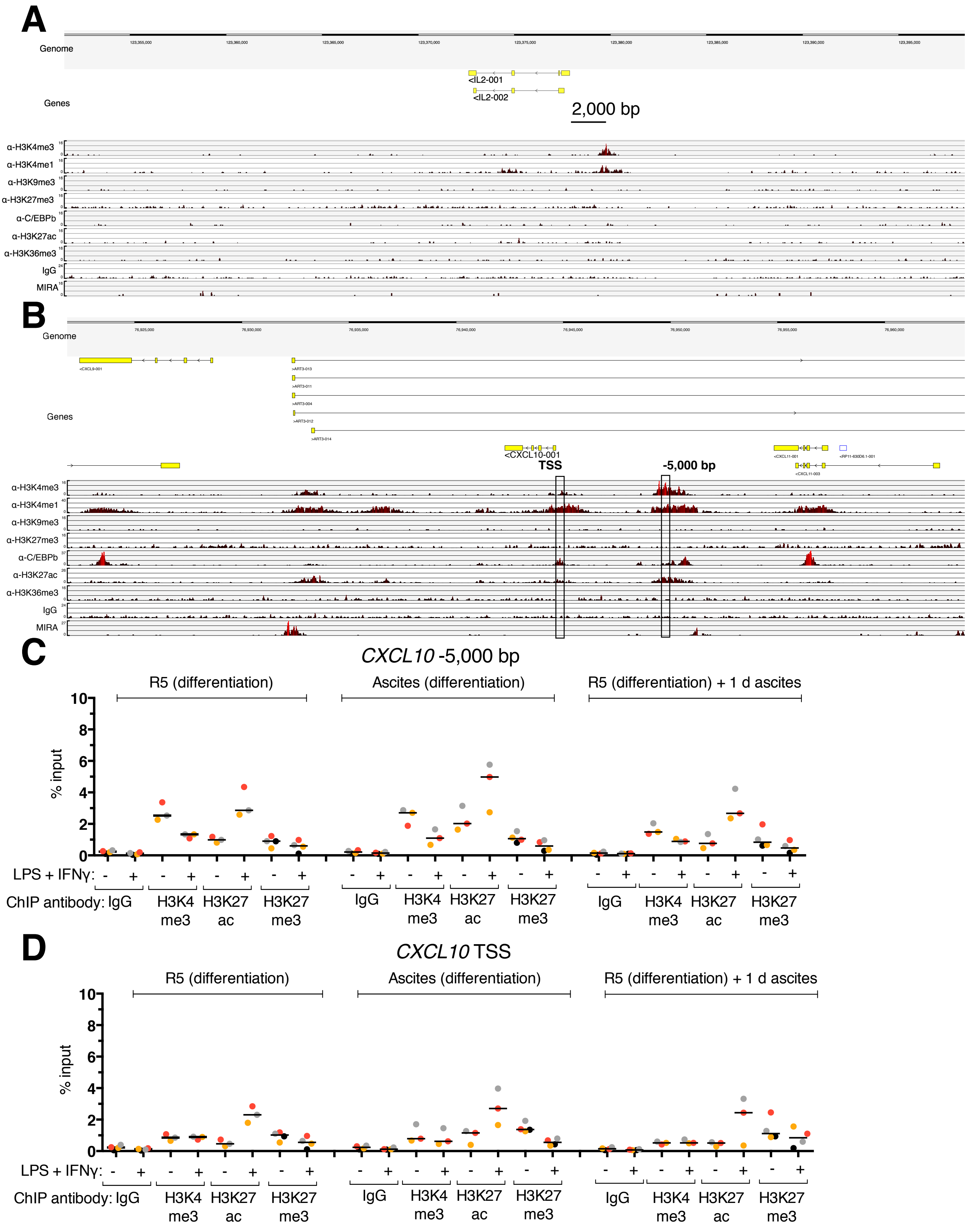

Figure S9: Chromatin marks at the *IL2* and *CXCL10* loci in TAMs and MDMs.

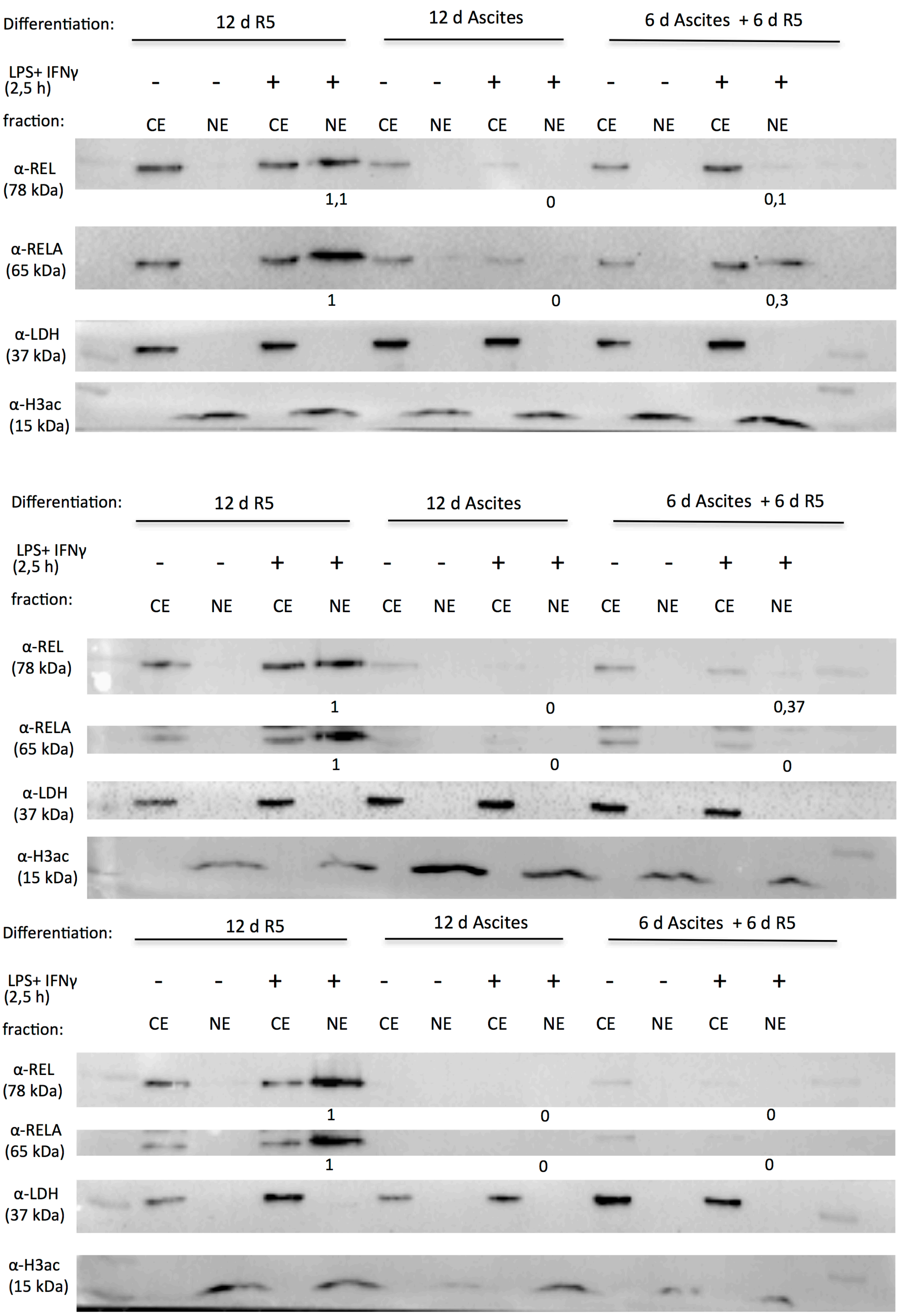

Figure S10: Original immunoblot images used for compilation of fig. 3.

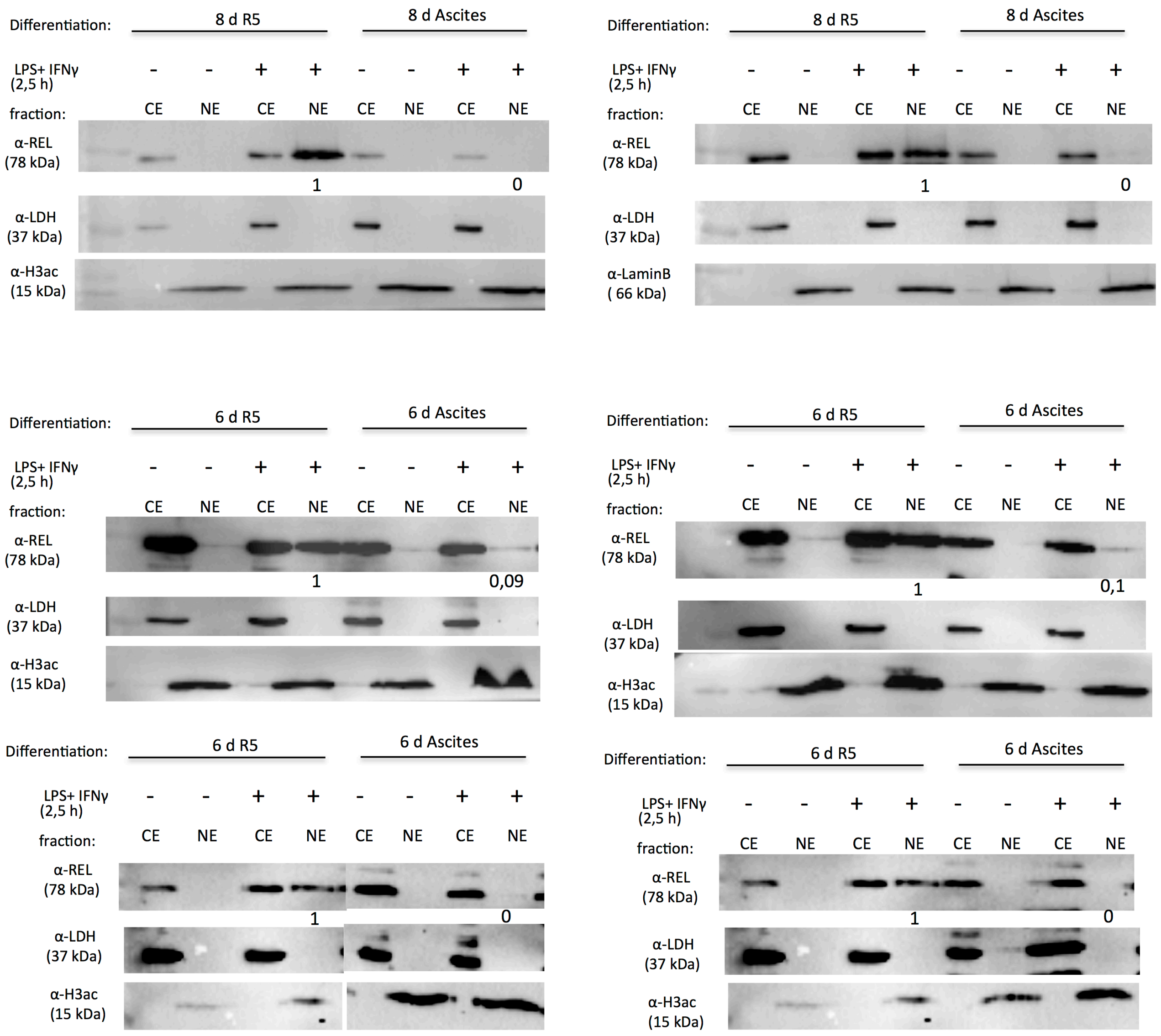

Figure S10: Original immunoblot images used for compilation of fig. 3.

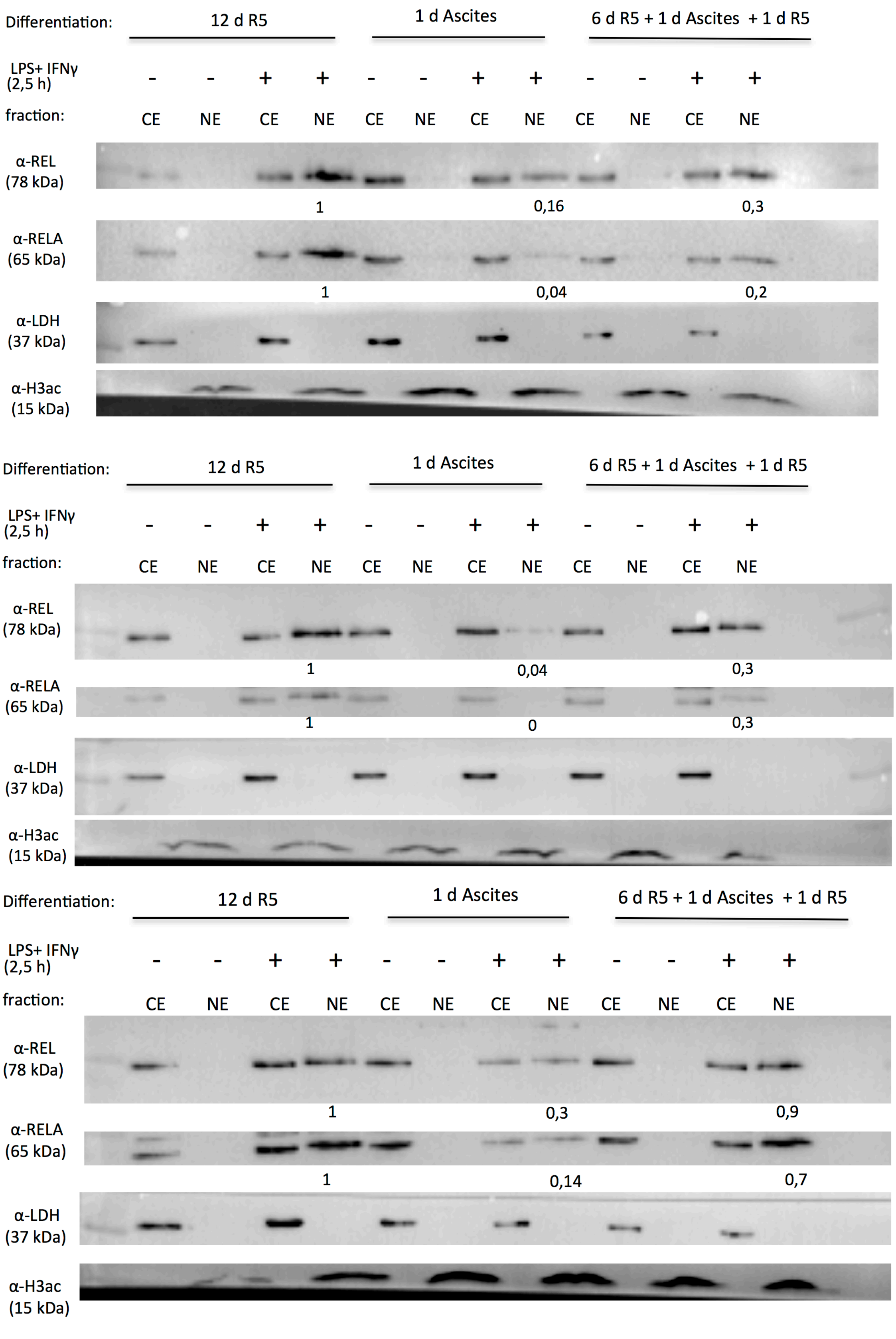

Figure S10: Original immunoblot images used for compilation of fig. 3.

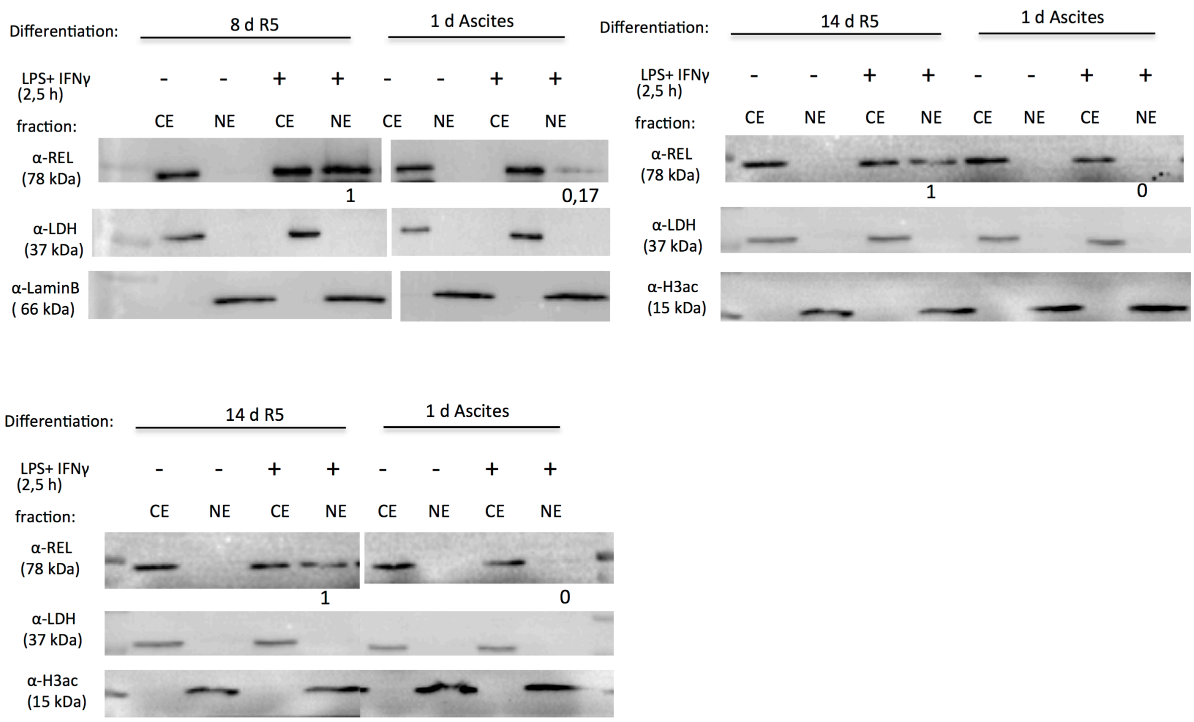

Figure S10: Original immunoblot images used for compilation of fig. 3.
